# Faf1 accelerates p97-mediated protein unfolding by promoting ubiquitin engagement

**DOI:** 10.1016/j.celrep.2026.117393

**Published:** 2026-06-02

**Authors:** Zengwei Liao, Connor Arkinson, Andreas Martin

**Affiliations:** 1 California Institute for Quantitative Biosciences, University of California, Berkeley, Berkeley, CA 94720, USA; 2 Department of Molecular and Cell Biology, University of California, Berkeley, Berkeley, CA 94720, USA; 3 Howard Hughes Medical Institute, University of California, Berkeley, Berkeley, CA 94720, USA; 4 Lead contact

## Abstract

P97/VCP is a protein unfoldase of the AAA+ ATPase family that plays essential roles in numerous processes, including ER-associated degradation and DNA replication. For unfolding of proteins modified with K48-linked ubiquitin chains, p97 works with the heterodimeric cofactor Ufd1-Npl4, and the cofactor Faf1 was shown to enhance this activity during replisome disassembly by unknown mechanisms. Here, we employ an *in vitro* reconstituted system with human components for biochemical experiments, FRET-based assays, and cryo-EM structure determination to reveal that Faf1 generally accelerates ubiquitin-dependent substrate processing by promoting the unfolding of an initiator ubiquitin and its engagement by the ATPase. Faf1 thereby uses its p97-bound C-terminal UBX domain to anchor a long helix that braces Ufd1’s UT3 domain and stabilizes Ufd1-Npl4 for ubiquitin unfolding. Our findings demonstrate how p97 works simultaneously with several cofactors to facilitate the unfolding of ubiquitinated proteins, indicating more complex regulatory mechanisms than for the simpler yeast Cdc48.

## In brief

Faf1 was previously shown to accelerate unfolding of poly-ubiquitin-tagged substrates by the p97 unfoldase and its cofactors Ufd1/Npl4. Here, structure-function studies by Liao et al. reveal the underlying mechanisms and how Faf1 stabilizes Ufd1 for unfolding of an initiator ubiquitin, followed by p97 insertion as first steps of substrate processing.

## Graphical Abstract

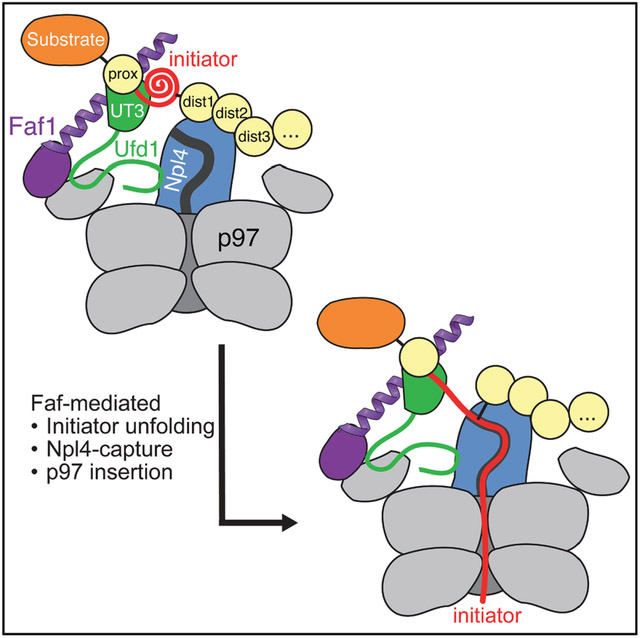

## INTRODUCTION

The mammalian valosin-containing protein (VCP) or p97 (Cdc48 in yeast) is an essential AAA+ (ATPases associated with various cellular activities) protein unfoldase that plays roles in many cellular processes, such as endoplasmic-reticulum-associated degradation (ERAD), endosomal trafficking, membrane fusion, DNA damage response, and DNA replication.^1-6^ A major function of p97 is to extract proteins from macromolecular complexes or membranes and unfold them prior to degradation by the 26S proteasome.^7^ Many of p97’s substrates are therefore marked with polyubiquitin chains. Mutations in p97 are for instance linked to cardiovascular and neurodegenerative diseases, including multisystem proteinopathy (MSP), a fatal disease with various clinical pathologies that affect the brain, muscle, and bone.^8-10^

P97 is a homo-hexamer with each protomer containing an N-terminal domain (NTD) and tandem ATPase domains, D1 and D2, that in the hexamer form stacked rings. Many cofactor proteins bind p97 to contribute various activities or regulate its function, including substrate recruitment, engagement, unfolding, and deubiquitination.^11^ Cofactors thereby use ubiquitin regulatory X (UBX/UBXL) domains or linear sequences such as VCP-binding motifs (VBMs), VCP-interacting motifs (VIMs), or SHP motifs (also called BS1, binding segment 1) to interact with p97’s NTD, while others bind the C-terminal tail of p97 through a PUB (peptide:N-glycanase and UBA- or UBX-containing proteins) or PUL (PLAP, Ufd3p, and Lub1p) domains. One of the most well-studied p97 cofactors is the heterodimeric Ufd1-Npl4 (UN) complex, which binds on top of the p97 hexamer, recruits ubiquitinated substrates, and initiates their unfolding, with specificity for K48-linked ubiquitin chains.^12,13^ Npl4 interacts through its UBXL and zinc-finger domains with p97’s NTD and D1 domains, while Ufd1 contacts Npl4 through a small interface and is highly flexible, which so far prevented its visualization by cryo-EM.^12^ Structural and functional analyses of UN-bound Cdc48 indicate that an initiator ubiquitin in the substrate-attached ubiquitin chain gets unfolded by UN in an ATP-independent manner and binds in a hydrophobic groove on Npl4 to insert its N terminus into the Cdc48 central channel. The Cdc48 motor then utilizes ATP hydrolysis to engage and pull on the initiator ubiquitin, unfold and translocate any proximal ubiquitin moieties in the chain, and thread the attached substrate protein itself.^12,14^ The ubiquitin moiety immediately distal to the initiator was observed to interact with parts of Npl4 and Ufd1, while the consecutive next two ubiquitins bind on top of the so-called tower domain of Npl4^12,15^ and increase substrate affinity during recruitment.^14^ Importantly, the ubiquitin moiety on the proximal, substrate-facing side of the initiator binds Ufd1’s UT3 domain, which appears to determine the K48-ubiquitin linkage specificity of Cdc48-UN and is critical for insertion of the initiator ubiquitin into the ATPase motor.^14^

Interestingly, only the proximal ubiquitin(s) and the substrate appear to get unfolded and threaded, whereas ubiquitins on the distal side of the initiator may not be unfolded.^14,16-18^ Release of the ubiquitinated substrate from the motor after unfolding therefore represents the rate-limiting step for both Cdc48-UN and p97-UN, at least in the absence of other cofactors or deubiquitinases.^14,18^ Furthermore, without any additional cofactors, p97-UN is far less efficient in substrate unfolding than Cdc48-UN and requires much longer ubiquitin chains,^19,20^ suggesting more complex regulatory mechanisms for substrate recruitment and processing.

In humans, there is an expanded family of p97 cofactors, but it remains unclear how, when, or which of these cofactors work together. Many of them may act independently, compete for the same binding sites on p97, or function only in the context of specific substrates. However, recent insights into replisome disassembly showed that fas-associated factor 1 (Faf1) works with p97-UN to enhance the unfolding of MCM complexes and their removal from DNA, potentially through lowering the threshold for the ubiquitin-chain length.^19^ Interestingly, in addition to replisome disassembly,^19,21^ Faf1 has been reported to play significant roles in other pathways, including immune response^22,23^ and aggresome clearance,^24^ indicating possible generic roles in multiple p97-dependent processes.

**Figure 1.**
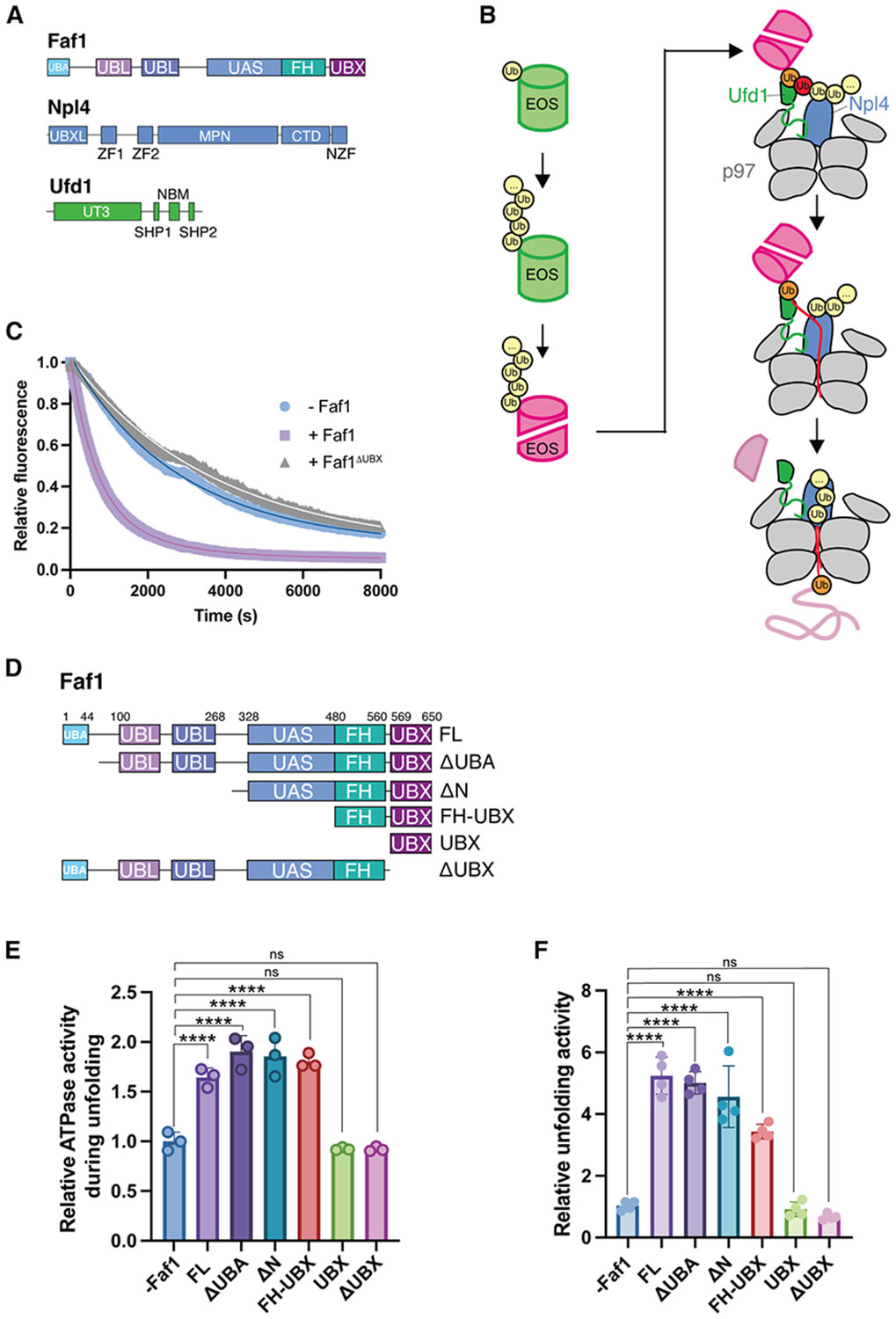
Faf1 accelerates the unfolding of ubiquitinated substrates (A) Schematics of domain architectures for human Faf1, Npl4, and Ufd1. (B) Left: overview of substrate preparation. mEos3.2 fused to mono-ubiquitin was poly-ubiquitinated and photoconverted by UV irradiation to red Eos with cleaved backbone. Right: the substrate-unfolding assay monitors the loss of red Eos fluorescence. An initiator ubiquitin (red sphere), the proximally neighboring ubiquitin (orange sphere), and ubiquitins on the distal side (yellow spheres) bind to the UT3 domain of Ufd1 (green) and the top of Npl4 (blue), before the initiator ubiquitin is unfolded, captured by Npl4, and inserted into p97, followed by unfolding and threading of the entire proximal part of the chain as well as the Eos substrate. Ubiquitin moieties located on the distal side of the initiator do not get unfolded by p97. (C) Traces for the loss of red Eos fluorescence upon unfolding by p97-UN in the absence and presence of Faf1. All traces represent the averages of three independent measurements that were normalized to the starting fluorescence value. Solid lines show the single-exponential fits. (D) Schematics of Faf1 truncation mutants used in biochemical experiments. (E) Relative ATPase activities of p97-UN during unfolding of ubiquitinated green Eos in the absence of Faf1 (normalized to 1) and the presence of full-length or truncated Faf1. Shown are the mean values and standard deviations of the mean for three technical replicates. Statistical significance was calculated using a one-way ANOVA test: ****p < 0.0001; ns, *p* > 0.05. (F) Relative substrate-unfolding activities of p97-UN in the absence of Faf1 (normalized to 1) and in the presence of saturating (2 μM) full-length or truncated Faf1. Shown are the mean values and standard deviations of the mean for four technical replicates. Statistical significance was calculated using a one-way ANOVA test: ****p < 0.0001; ns, *p* > 0.05.

Faf1 is a multidomain protein (Figure 1A) that uses its C-terminal UBX domain for binding to p97’s NTD,^25^ potentially interacts with ubiquitin chains through its N-terminal UBA (ubiquitin-associated) domain,^26^ and contacts other chaperones, like Hsp70, through one of its two UBL domains.^27^ However, the mechanism for Faf1-mediated enhancement of substrate unfolding by p97-UN remains unclear.

Here, we *in vitro* reconstitute p97-UN unfolding of model substrates with K48-linked ubiquitin chains and reveal that Faf1 has a generic function by facilitating initiation, increasing substrate capture, and thus accelerating processing. Our FRET-based studies show that Faf1 specifically accelerates initiator-ubiquitin insertion into p97, with rates that are comparable to those of Cdc48-UN. Interestingly, p97 subsequently unravels the mEos model substrate much more slowly than Cdc48, suggesting mechanical unfolding as the rate-limiting step for p97. Using cryo-EM single-particle analysis, we observe the p97-UN-Faf1 complex with a ubiquitin chain during unfolding initiation. Faf1’s C-terminal UBX domain binds to the NTD of p97 and stabilizes the SHP motif of Ufd1, which is key to Faf1 recruitment. A helix preceding the UBX domain reaches up and braces Ufd1’s UT3 domain. This UT3 domain shows at least two bound ubiquitin moieties, with one being linked to the unfolded initiator ubiquitin that interacts with Npl4’s hydrophobic groove and reaches into the p97 hexamer. Site-specific mutagenesis combined with our substrate-unfolding and FRET-based assays confirmed that Faf1’s helix interactions with the UT3 domain of Ufd1 allow for more efficient initiator-ubiquitin unfolding and insertion into p97. Finally, we show that this mechanism is conserved for the membrane-associated cofactor Faf2, which plays critical roles in ERAD.^28,29^ Faf1 and Faf2 promote a more productive complex between UN, p97, and ubiquitinated substrates that allows higher efficiency in ubiquitin insertion and unfolding initiation, which indicates how those cofactors may generically support the numerous cellular functions of p97-UN.

## RESULTS

### Faf1 stimulates unfolding activity of p97-UN

To test whether Faf1 can generally accelerate the unfolding of ubiquitinated substrates by p97-UN, we utilized a photoconverted, red-fluorescent, and thus irreversibly unfolding mEos3.2 model substrate with a covalently fused mono-ubiquitin that was enzymatically extended into long (>8 moieties) K48-linked polyubiquitin chains (Figure 1B; Figures S1A and S1B).^30,31^ Human p97, UN, and Faf1 were recombinantly expressed and purified from *Escherichia coli*. Reconstituted Eos-unfolding experiments under single-turnover conditions (i.e., enzyme at saturating concentrations and in excess over substrate) in the absence or presence of Faf1 showed that Faf1 accelerates p97-UN-mediated substrate processing by ~5-fold, from τ_Eos unfold_ (−Faf1) = 4727 ± 688 s to τ_Eos unfold_ (+Faf1) = 902 ± 76 s (Figure 1C; Figure S1C). These results are overall consistent with previous findings about the Faf1 effects on MCM-complex disassembly,^19^ but, importantly, also demonstrate a role in accelerating unfolding of ubiquitinated substrates in general.

### A helix-UBX domain fragment of Faf1 is sufficient for facilitating substrate unfolding

To assess which parts of the multi-domain Faf1 are responsible for the stimulation of substrate unfolding by p97, we generated several truncated variants (Figure 1D; Figure S1D) and tested their effect on both substrate processing and ATP hydrolysis (Figures 1E and 1F). Faf1 alone, in the absence of UN and substrate, did not increase p97’s low basal ATPase activity (Figure S1E). However, Faf1 stimulated ATP hydrolysis ~1.7-fold in the presence of substrate and UN (Figure 1E), suggesting that it either accelerates the motor during threading and unfolding or it facilitates substrate engagement, thereby increasing the fraction of actively substrate-processing p97, with ATP hydrolysis above idling.^18^ While Faf1’s UBA and UBL domains were dispensable for p97 activation, deletion of its UBX domain abolished Faf1’s stimulatory effects (Figures 1E and 1F), which is consistent with previous observations for p97-mediated replisome disassembly in the presence of Faf1.^19^ Faf1’s two UBL domains were suggested to function in binding other chaperones, such as Hsp70, and thus may play context-specific roles,^23,27^ similar to the UAS domain that appears to bind fatty acids.^32^ We found that a minimal C-terminal fragment consisting of the FH and UBX domain (FH-UBX) leads to a stimulation of p97’s unfolding activity that is comparable to that of full-length Faf1, whereas the UBX domain alone was insufficient for stimulation (Figure 1F).

### Faf1 does not enhance the activity of p97 bound to yeast UN

Since there are clear differences between human and yeast UN, we wondered whether human UN is more dynamic and therefore depends on Faf1 to form a productive complex for unfolding initiation. Interestingly, yeast UN (yUN) can support p97 unfolding of the ubiquitinated-Eos substrate at a rate of τ_Eos_ = 26.7 ± 2.4 min, which is ~3.5-fold higher than the rate in the presence of human UN (hUN) without Faf1 (Figure S1F) and demonstrates the functional conservation of the AAA+ motors. The addition of full-length Faf1 did not further stimulate the unfolding activity of p97-yUN but in fact reduced it (Figure S1F), potentially because Faf1’s UBL domains bind to the tower ubiquitin-binding sites of the yeast Npl4. We therefore used the FH-UBX domain fragment of Faf1 and observed an unfolding activity that was comparable to that of p97-yUN (Figure S1F). yUN may intrinsically encode for more productive ubiquitin unfolding and insertion into Cdc48 or p97, whereas hUN is potentially more dynamic and depends on additional interactions such as Faf1’s FH-UBX-domain portion.

### Cryo-EM reveals a pre-initiation state with flexibly bound Npl4

For gaining structural insights into Faf1’s stimulating effects on p97 unfolding activity, we aimed to capture a substrate-engaged p97-UN-Faf1 complex. In a first attempt, we initiated the unfolding reaction with ATP, then switched through buffer exchange to the non-hydrolyzable analog ATPγS in order to stall substrate-engaged complexes (Figures S2A and S2B), and analyzed the sample by cryo-EM (Figure S3; Table S1). However, single-particle analysis resolved only the p97 hexamers with no defined density for most of Faf1, the UN complex, or the substrate, likely due to high flexibility and conformational heterogeneity. Symmetry expansion and focused classification of individual p97 promoters with a mask on the NTD resolved Faf1’s UBX domain bound to the NTD (Figures S4A and S4B), which is in agreement with previous crystal structures,^33,34^ and just parts of the preceding long helix extending up from the NTD.

We therefore turned to a different experimental setup that used p97-UN with the FH-UBX fragment of Faf1 and long unanchored ubiquitin chains instead of the ubiquitinated model substrate, in an attempt to reduce conformational heterogeneity. We reconstituted these components in the presence of ATP to promote functional complex states during unfolding initiation. Cryo-EM analysis of this sample revealed a minor fraction of particles in a potential apo or pre-initiation (PI) state, in which Npl4 is resolved on top of the p97 hexamer, but there is no indication of ubiquitin, and only the NTD-bound SHP motifs and UBX domains are visible for Ufd1 and Faf1, respectively (Figures S5-S7). Npl4 is at lower resolution and apparently flexibly bound, using its UBX domain to interact with the NTD of protomer E and its zinc finger 1 (ZF1) to contact the D1 ATPase domain of protomer F in the p97 hexamer, while ZF2 does not interact with an ATPase domain and is unresolved (Figure S7B). The planar D1 ring in this PI state has protomers A, B, and F occupied with ADP and protomers C–E bound to ATP, whereas the entire D2 ring is bound to ADP (Figure S7E), similar to what was observed for the D2 ring in Cdc48-UN during initiation.^15^ In this state, the “Npl4 loop” (residues 425–439) is positioned right above the entrance to the p97 channel, in a conformation that would likely inhibit initiator-ubiquitin insertion into the motor (Figure S7A). This also resembles the previously described ubiquitin-bound Cdc48-UN complex, in which the Npl4 loop adopted a similar position and ubiquitin was absent from the Npl4’s groove.^15^ However, in contrast to our structure of human p97-UN, the yeast complex showed ubiquitins bound to the top of Npl4, which interacted through both ZF1 and ZF2 with the D1 ring of p97.

### Faf1 braces the UT3 domain of Ufd1 for initiator unfolding

**Figure 2.**
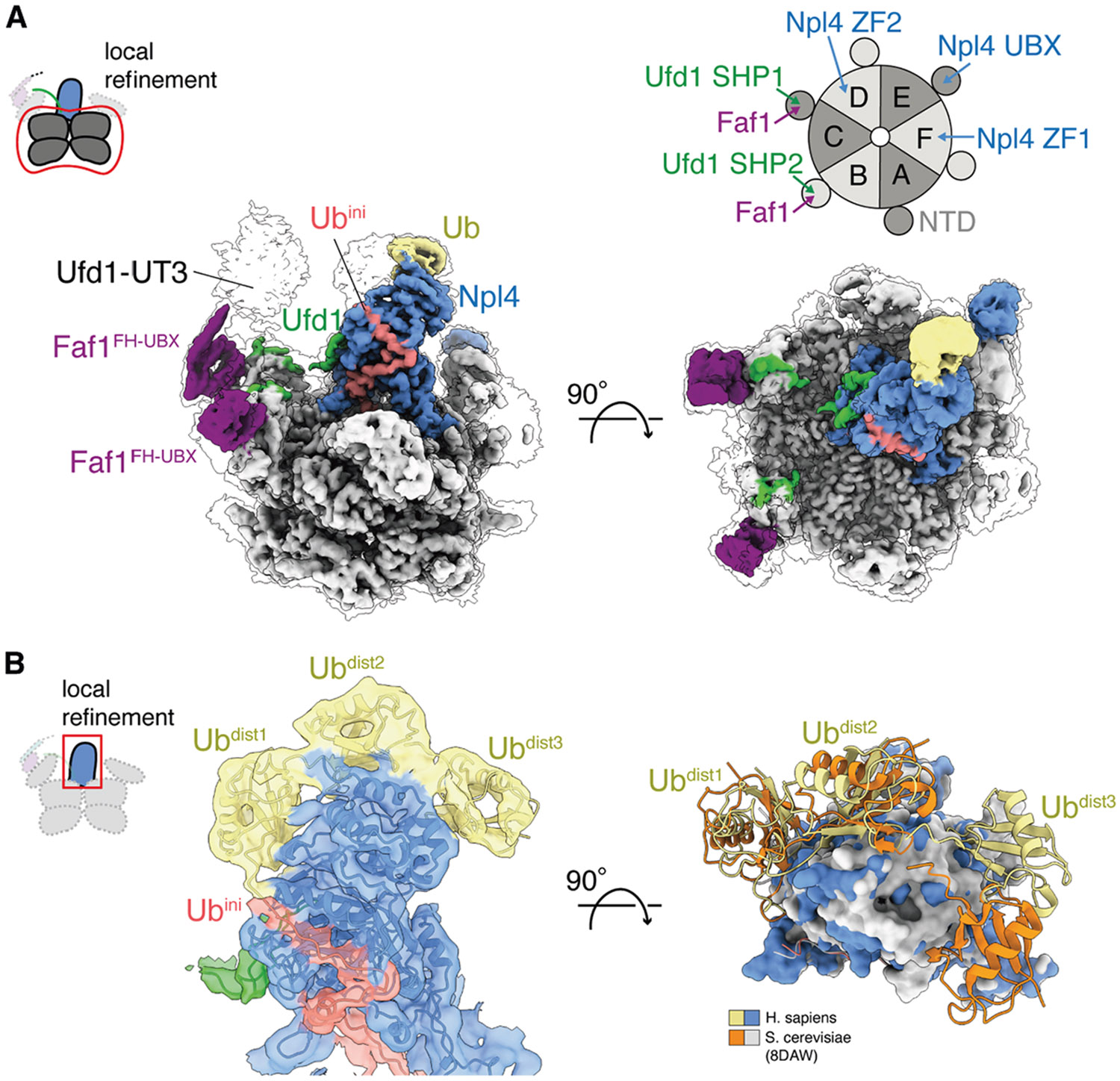
Cryo-EM structure of the poly-ubiquitin-bound p97-UN-Faf1^FH–UBX^ initiation complex (A) Local refinement of the ATPase motor reveals Npl4 (blue) on top of the p97 hexamer (gray), with a bound initiator ubiquitin (Ub^ini^, salmon) reaching into the D1 ATPase ring. The SHP1 and SHP2 motifs of Ufd1 (green) occupy two neighboring NTDs of p97 together with two Faf1 UBX domains (purple). A lower threshold map is shown transparent to visualize Ufd1’s UT3 domain and extra density on Npl4. Top right: schematic of the p97 hexamer with protomers A–F, illustrating the binding positions of Npl4 (ZF1; ZF2, UBX), Ufd1 (SHP1, SHP2), and Faf1 on the D1 ATPase ring. (B) Left: locally refined cryo-EM density focused on Npl4 and its interacting ubiquitins. Three distal ubiquitins (Ub^dist1^-Ub^dist3^, yellow) are resolved and bound to top of Npl4 (blue), with Ub^ini^ (salmon) in the hydrophobic groove and the Npl4-binding motif of Ufd1 (green) visible at the base. Right: atomic model for the human Npl4 (blue surface) in complex with Ub^dist1^-Ub^dist3^ (yellow ribbons) overlayed with the structure of the ubiquitin-bound yeast Cdc48-UN (Npl4 as white surface, Ub^dist1^-Ub^dist3^ as orange ribbons; PDB ID: 8DAW).

The majority of particles in our dataset represented initiation-state (I state) complexes, with p97 bound to UN, Faf1, and a ubiquitin chain during motor insertion. Local refinement of the p97-Npl4 portion revealed Npl4 stably bound to the D1 ring, with ZF1 and ZF2 contacting protomers F and D, respectively (Figure S7C). The D1 ring contains three ADP and three ATP and thus has an identical nucleotide occupancy as in the pre-initiation state (Figure S7E), but protomer E and F are noticeably shifted downward (Figure S7D). The entire D2 ring is again filled with ADP (Figure S7E). Similar to the initiation state previously observed for Cdc48-UN,^12^ an unfolded initiator ubiquitin (Ub^ini^) is bound to Npl4’s groove and reaches with its N terminus into the p97 central channel, with Lys11 as the last resolved residue positioned near the bottom of the D1 ATPase ring (Figure 2A; Figure S7D). Through further focused classification with a mask on Npl4, we were able to identify a sub-state that showed the three ubiquitin moieties linked to Ub^ini^ on its distal side, Ub^dist1^, Ub^dist2^, and Ub^dist3^, bound on top of Npl4 (Figure 2B). While Ub^dist2^ is in a stable position, Ub^dist1^ shows higher heterogeneity between subclasses, and Ub^dist3^ is visualized in only a few classes, located in a different position compared to previous structures of the ubiquitin-bound yeast Cdc48-UN (Figure 2B).^15^

**Figure 3.**
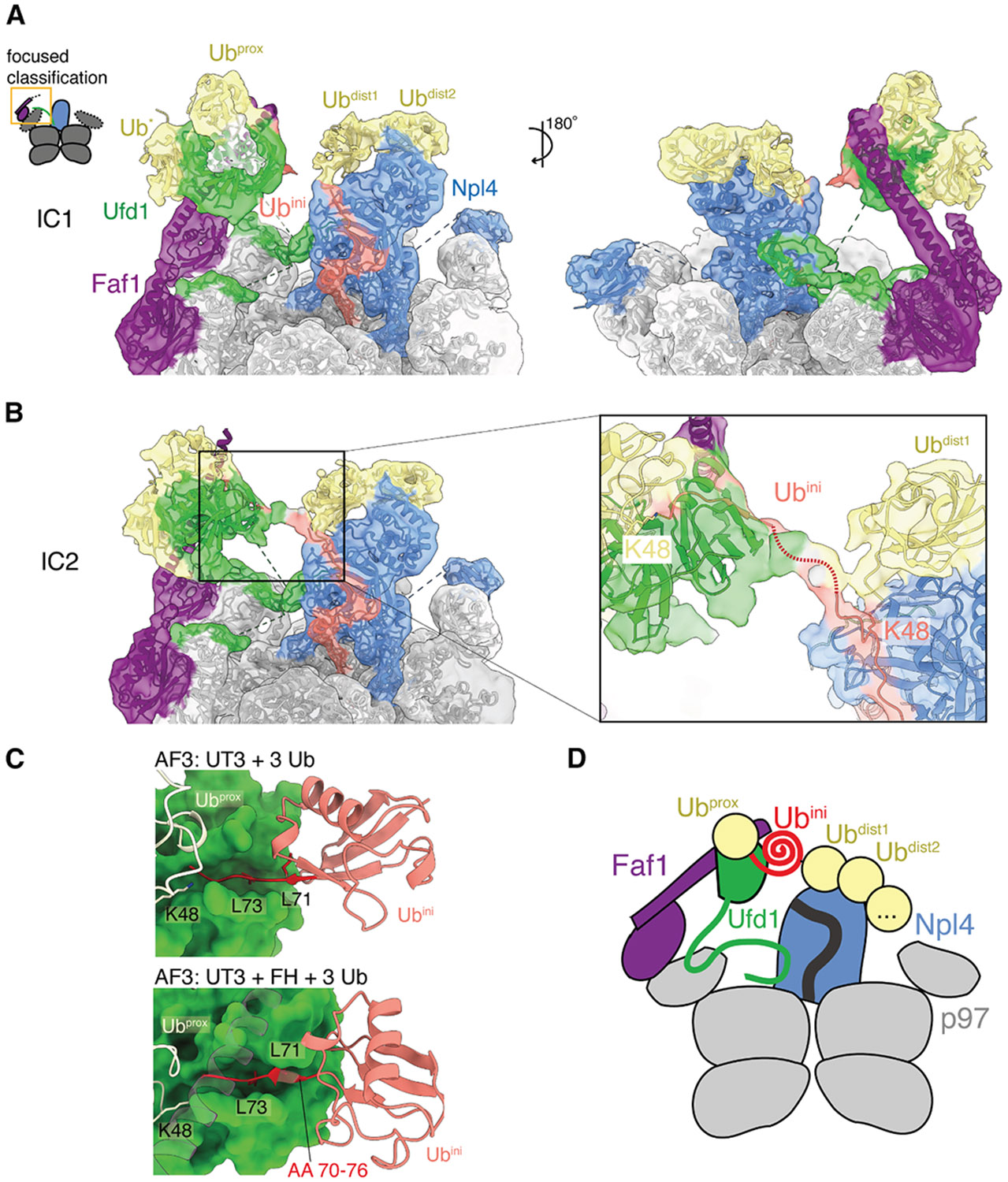
Faf1 braces the ubiquitin-bound UT3 domain of Ufd1 for initiation (A) Cryo-EM density map of the initiating conformation 1 (IC1) is shown in front and back view, with p97 in gray, Npl4 in blue, Ub^ini^ in salmon, the proximal and distal ubiquitins (Ub^prox^ and Ub^dist^) in yellow, Ufd1 in green, and Faf1^FH–UBX^ in purple. (B) IC2 density map with the inset showing connecting density between the Npl4-bound Ub^ini^ and K48 of Ub^prox^ and a red dashed line indicating the potential path of the unfolded Ub^ini^ backbone. (C) Top: AlphaFold model of Ufd1’s UT3 domain with three ubiquitins suggests an additional binding site accommodating the last seven residues (red) of Ub^ini^ (salmon). Bottom: inclusion of Faf1’s FH in the AlphaFold predictions leads to a different conformation of Ub^ini^’s C terminus, with residues 70–72 forming a small beta sheet with the UT3 domain and L71 as well as L73 docking into hydrophobic pockets. (D) Schematic model for a putative pre-initiation state that precedes IC1 and IC2, in which Ub^dist^ moieties are bound to the top of Npl4, Ub^prox^ interacts with Ufd1’s UT3 domain, and Ub^ini^ is destabilized by binding of its C-terminal residues to UT3, mediated by Faf1.

We also observed two Faf1 UBX domains, bound to the NTDs of p97 protomers B and C, and additional density extending from these domains toward Npl4. To improve the resolution and provide insight into the ubiquitin chain on the proximal side of the Npl4-bound unfolded Ub^ini^, we used a mask around the NTD of protomer C, Faf1’s UBX-helix portion, and the space toward Npl4 for further 3D classifications (Figure 3). Global refinement of these classes revealed improved density for the Faf1 helix on p97 protomer C and the interacting density, which was particularly prevalent in two classes that we name initiating conformation 1 and 2 (IC1 and IC2; Figures 3A and 3B). For identifying this additional density in contact with the Faf1 helix, we used AlphaFold predictions of a p97-UN-Faf1-ubiquitin complex, which indicated direct interactions of the Faf1 helix with the UT3 domain of Ufd1 (Figure 3A). Indeed, in IC1, we could model the structure of the UT3 domain with two bound ubiquitins into the extra density, and we name these ubiquitins Ub^prox^ and Ub*. The interaction sites of Ub^prox^ and Ub* on the UT3 domain were previously described in NMR-binding studies of polyubiquitin and monoubiquitin, respectively.^35^ Notably, the C terminus of Ub^prox^ is not in proximity of Ub*’s K48, indicating that these ubiquitins are not linked, but it points toward an additional density that potentially represents a third, poorly resolved ubiquitin on the UT3 domain (Figure S8).

In IC2, we also identified Ufd1’s UT3 domain with slightly less well-resolved Ub^prox^ and Ub* moieties but noticed a shift in its position relative to Npl4 (Figure 3B). Furthermore, there was continuous linking density from the UT3 domain to the unfolded Ub^ini^ in the Npl4 groove. Interestingly, AlphaFold models predict another ubiquitin binding site on the UT3 domain, in which ubiquitin’s G76 points toward K48 of Ub^prox^, the preceding C-terminal residues form a small beta-sheet with the UT3 domain, and Leu71, normally part of ubiquitin’s hydrophobic core, docks into a hydrophobic pocket of UT3 (Figure 3C). We previously found for yeast Cdc48-UN that the ubiquitin moiety proximal to the initiator binds to the UT3 domain and that this interaction is responsible for the K48-linkage selectivity of ubiquitin unfolding by Cdc48-UN.^14^ We therefore reasoned that the AlphaFold-predicted, C-terminally bound ubiquitin on the UT3 domain represents the initiator-ubiquitin moiety prior to its unfolding. Although the linking density between UT3 and Npl4 is too low in resolution to unambiguously model the C terminus of Ub^ini^, it directly connects the Npl4-bound Ub^ini^ with K48 of Ub^prox^ on the UT3 domain, supporting our hypothesis that the C terminus of the unfolded initiator ubiquitin binds to the UT3 domain in the AlphaFold-predicted position. This is also consistent with a recent study that investigated this binding site for ubiquitin’s C-terminal tail on the UT3 domain through X-ray crystallography and mutagenesis.^36^

In both IC1 and IC2, the FH helix of the Faf1 copy on the p97 protomer C contacts the UT3 domain and Ub^prox^ (Figure 3). Direct contact between FH and ubiquitin at the UT3 domain is supported by photo-induced crosslinking experiments, in which we used Cy3-labeled ubiquitin chains and Cy5-labeled Faf1 FH-UBX fragment with benzophenylalanine (BPA) incorporated at position 501 to show that Faf1’s ubiquitin interaction depends on the presence of p97 and UN (Figure S9). These findings are also consistent with recent other reports of interactions between FH and ubiquitin.^37^ Faf1 likely serves as a stabilizing scaffold that bridges p97’s NTD with the UT3 domain, positioning UT3 to facilitate the unfolding of Ub^ini^, its capture by the Npl4 groove, and insertion into the p97 motor. Indeed, performing AlphaFold predictions of ubiquitin-UT3 interactions with and without FH suggests that the presence of the Faf1 helix promotes a different conformation of Ub^ini^’s last seven residues on the UT3 domain, which may have an effect on Ub^ini^ binding and unfolding (Figure 3C). It is conceivable that in an initial binding stage. the ubiquitin chain drapes over the p97-mounted UN cofactor, with Ub^prox^ and the C terminus of the Ub^ini^ interacting with Ufd1’s UT3 domain, while the consecutive Ub^dist1^, Ub^dist2^, and Ub^dist3^ bind on top of Npl4 (Figure 3D). Interaction of Ub^ini^’s C terminus with the UT3 domain, facilitated by Faf1, is expected to destabilize Ub^ini^ and induce its unfolding for binding to the Npl4 groove.

Interestingly, from the UBX domain of the protomer-B-bound second Faf1, we observed low-resolution density extending up to the opposite side of the UT3 domain, such that UT3 appears to be braced by the helices of two Faf1, possibly for additional stability or positional control (Figure S8A). Individual subclasses show the helix of the second Faf1 differentially resolved, depending on the movement of the UT3 domain relative to Npl4 (Figure S8B), which further supports our model for Faf1-mediated UT3-domain positioning and a potential role in initiator-ubiquitin unfolding.

The number and location of Faf1 molecules on the p97 hexamer seems thereby determined by the SHP motifs of Ufd1. Using a locally refined map from our first cryo-EM dataset together with AlphaFold predictions, we identified Ufd1’s SHP1 motif bound to the NTD of p97 protomer C, with its L235 anchored in a hydrophobic hole of the NTD (Figures 3C and 3D; Figure S10)^38,39^ and a linking density to Npl4, while the C-terminal SHP2 motif is bound to the NTD of protomer B (Figures 3C and 3D). Although it cannot be ruled out that the presence of two Faf1 copies is a consequence of our *in vitro* reconstitution, the correlation between Faf1-UBX-domain binding and Ufd1-SHP-motif binding to the same NTDs of p97 suggests a functional and physiological relevance.

### Faf1 promotes ubiquitin insertion and engagement by p97

**Figure 4.**
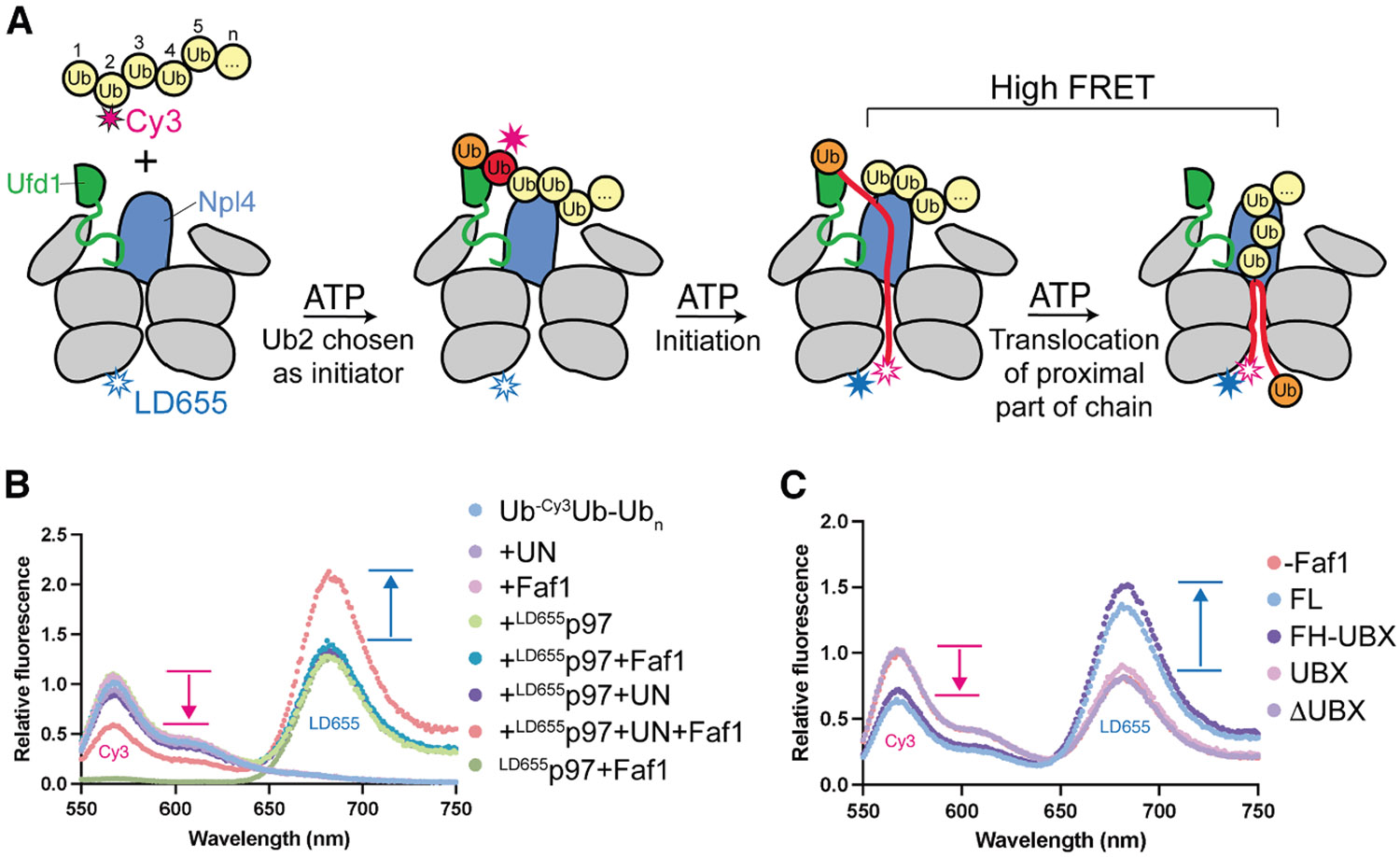
FRET-based unfolding-initiation assay (A) Experimental design for the initiation assay, using unanchored poly-ubiquitin chains (>10 moieties) labeled with Cy3 (red star) on the N terminus of the second ubiquitin and p97 labeled with LD655 (blue star) near the exit of the processing channel. UN choosing ubiquitin 2 as the initiator (top) leads to an immediate FRET-signal change after insertion of that ubiquitin moiety. (B) Normalized fluorescence emission spectra after excitation at 480 nm for ubiquitin chains with Cy3 attached to ubiquitin 2 (Ub-^Cy3^Ub-Ub_n_) in the absence and presence of LD655-labeled p97 (^LD655^p97), UN, and Faf1 or for ^LD655^p97 with Faf1 alone. Arrows indicate the changes in Cy3- and LD655-fluorescence intensities upon unfolding initiation. (C) Normalized fluorescence emission spectra after Cy3 excitation for samples with Ub-^Cy3^Ub-Ub_n_, ^LD655^p97, and UN in the absence or presence of different Faf1 truncation mutants. Arrows indicate the changes in Cy3- and LD655-fluorescence intensities upon unfolding initiation.

To test whether the FH-UT3 domain interaction is critical for the Faf1-mediated stimulation of substrate unfolding through a potential acceleration of initiator-ubiquitin unfolding and insertion, we employed an FRET-based initiation assay that we previously developed for Cdc48-UN.^14^ For this assay, the second ubiquitin in unanchored long (>10) ubiquitin chains was labeled on an engineered cysteine near its N terminus with a fluorescent donor dye (Cy3). P97 subunits were labeled with an acceptor dye (LD655) on the unnatural amino acid azido-phenylalanine (AzF) that was introduced in place of D592, located near the exit of the central channel (Figure 4A). Unfolding and threading of the donor-labeled ubiquitin is thus expected to bring it into proximity of the acceptor label and increase the apparent FRET efficiency. Control experiments confirmed that p97 with incorporated AzF and LD655 labels had unfolding activity, albeit at a reduced level compared to the unmodified enzyme (Figures S11A-S11C). Measurements of fluorescence spectra after mixing the Cy3-labeled ubiquitin chains with ^LD655^p97-UN and ATP in the absence of Faf1 showed no difference in donor or acceptor fluorescence compared to the isolated components, whereas the presence Faf1 caused significant reciprocal changes in fluorescence intensities that are indicative of FRET (Figure 4B). Faf1 thus appears to stimulate initiator-ubiquitin unfolding and insertion into the central channel, whereas UN alone is too inefficient for a considerable signal change over the time frame of this FRET assay, even though it can support slow substrate processing in our unfolding assay (Figure 1C).

It is important to note that the changes in donor and acceptor signals were stable for an extended amount of time (>10 min), suggesting that the ubiquitin chain either stalls in p97 or reaches a steady state of continuous unfolding and release, with the labeled second ubiquitin located inside or below the exit of the central channel and thus in proximity to the acceptor dye. For Cdc48-UN, initiator-ubiquitin unfolding and insertion was found to be fast (~3 s) even in the presence of ATPγS,^14^ whereas p97-UN required ATP hydrolysis for a signal change in our initiation assay (Figures S11D and S11E). It is possible that p97 depends on a combination of ATP- and ADP-bound subunits in the hexamer to adopt an appropriate spiral-staircase conformation for ubiquitin insertion. Alternatively, an initiator ubiquitin may be able to insert into ATPγS-bound p97, but the long chains necessary for efficient initiation by human p97-UN may strongly reduce the probability that the second, donor-labeled ubiquitin is chosen as the initiator in our assay. This model would also be consistent with our structural data, showing density for at least one additional ubiquitin besides Ub^prox^ bound to Ufd1’s UT3 domain. Initiation at position 3 or higher up in the chain would require ATP-hydrolysis-driven unfolding and translocation of proximal moieties to move the labeled ubiquitin 2 through the pore and into proximity of the acceptor for an FRET signal change (Figure S11F).

Using the double mutations W241A/R242E or E446K/Y447A in the ubiquitin-binding groove of Npl4,^12,14^ we confirmed that initiator-ubiquitin insertion into p97 still depended on its interaction with the Npl4 groove, even in the presence of Faf1 (Figure S11G). In agreement with our findings for substrate unfolding, FRET-based initiation assays with truncated Faf1 variants showed that the UBX domain is critical for p97 binding and that the minimal FH-UBX domain fragment is sufficient for accelerating ubiquitin insertion into the central channel of p97 (Figure 4C).

Despite the presence of Faf1, unfolding of the ubiquitinated Eos substrate by p97-UN is ~200 times slower than by Cdc48-UN (Figure 1C).^14^ To assess potential differences in the kinetics of unfolding initiation, we monitored the FRET changes upon labeled ubiquitin insertion into p97-UN-Faf1 in stopped-flow mixing experiments. Interestingly, ubiquitin insertion occurred with an apparent time constant of τinitiation = 5.1 ± 0.2 s (Figure S11H), only ~1.6-fold slower than for Cdc48-UN (τ_initiation_ = 3.2 ± 0.1 s).^14^ Notably, as described above, it is likely that a ubiquitin moiety distally located relative to the labeled ubiquitin 2 is chosen as the initiator, and additional unfolding and translocation of proximal ubiquitins is required for FRET signal development, such that the initiation time constant measured in our assay represent an upper bound. The velocity of substrate unfolding under single-turnover conditions is therefore not limited by slow initiation or the distinct ubiquitin-chain length requirements of p97-UN compared to Cdc48-UN but due to slow Eos unfolding itself. To confirm that p97 does not get stuck on the folded Eos domain, we used an Eos-Turquoise fusion substrate. The observed loss in Turquoise fluorescence confirmed that Eos is completely unfolded and threaded by p97, albeit very slowly (Figure S12). The significant difference in substrate-unfolding speeds between p97 and Cdc48 (τ_Eos unfold_ = 901.5 ± 75.6 s and τ_Eos unfold_ = 4.4 ± 0.1 s, respectively) may originate from the almost two orders of magnitude difference in ATP-hydrolysis activities (16 min^−1^ for p97 versus ~1,000 min^1^ for Cdc48 in the absence of UN and substrate^14,20^). Furthermore, p97 may have extra layers of negative regulation that could be encoded through sequence differences or additional cofactors. Assuming that Faf1 primarily facilitates the unfolding and insertion of the initiator ubiquitin, we can use the observed time constants for Eos processing in the absence versus presence of Faf1 [τ_Eos unfold_ (−Faf1) = 4727 ± 688 s and τ_Eos unfold_ (+Faf1) = 902 ± 76 s] together with the time constant for initiation with Faf1 from the FRET-based assay (τ_initiation_ = 5.1 ± 0.2 s) to estimate that Faf1 accelerates initiation by more than 700-fold. Subsequent unfolding of the Eos moiety in our model substrate occurs much more slowly and becomes rate-limiting, accounting for ~20% of the time spent on substrate processing in the absence of Faf1, which explains the maximum 5-fold overall acceleration by Faf1, despite its major effects on the efficiency and velocity of initiation.

### The stimulatory helix interaction with Ufd1’s UT3 domain is conserved for Faf2

**Figure 5.**
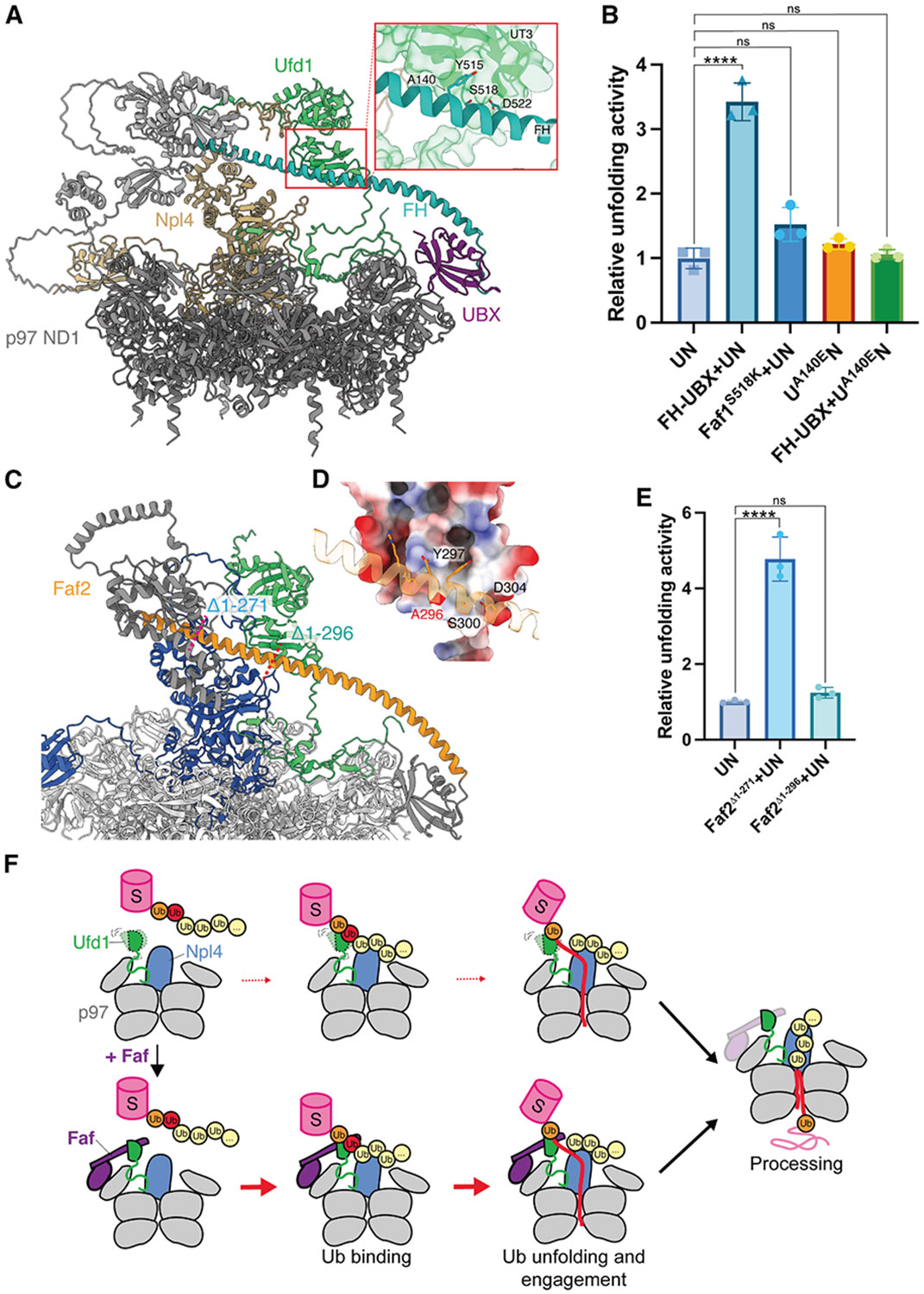
Faf1’s C-terminal helix facilitates initiator-ubiquitin unfolding and insertion into p97 (A) AlphaFold3 model for the complex between p97’s N-terminal and D1 ATPase domains (ND1, dark gray), Npl4 (tan), Ufd1 (green), and Faf1, with Faf1’s UBA, UBL, and UAS domain depicted in light gray, the long helix (FH) in turquoise, and the UBX domain in purple. The red box shows a zoom-in of the interface between Faf1’s long helix and Ufd1’s UT3 domain. (B) Relative mEos-substrate unfolding activities for p97 in the presence of wild-type UN, wild-type Faf1 FH-UBX fragment, UN with Ufd1 carrying an A140E mutation in its UT3 domain at the FH-binding interface, or Faf1 with a S518K mutation in its UT3-binding interface. Shown are the mean values and standard deviations of the mean for three technical replicates. Statistical significance was calculated using a one-way ANOVA test: ****p < 0.0001; ns, *p* > 0.05. (C) AlphaFold 3 model for the complex between p97’s ND1 portion (light gray), Npl4 (dark blue), Ufd1 (green), and Faf2, with the long helix shown on orange and the rest of Faf2 in dark gray. Positions for Faf2 N-terminal truncation mutants Δ1-271 and Δ1-296 are indicated by red dashed lines. (D) Close-up view of the interface between Ufd1’s UT3 domain and Faf2’s FH helix, with UT3 shown in surface representation and colored by electrostatic properties. (E) Relative mEos-substrate unfolding activities for p97-UN in the absence or presence of Faf2 N-terminal truncation variants. Shown are the mean values and standard deviations of the mean for three technical replicates. Statistical significance was calculated using a one-way ANOVA test: ****p < 0.0001; ns, *p* > 0.05. (F) Model for Faf-mediated engagement and unfolding of a ubiquitinated substrate (blue) by p97-UN. In the absence of a Faf cofactor, Ufd1’s UT3 domain may have higher flexibility that hampers the binding, unfolding, and motor engagement of the initiator ubiquitin (red sphere). Binding of Faf (purple) stabilizes the UT3 domain through its long helix that is anchored on its p97 NTD-bound UBX domain, facilitating unfolding initiation and overall substrate processing.

Truncation of Faf1’s FH abolished Faf1’s stimulatory effects on substrate unfolding activity (Figure 1F), consistent with our model that FH stabilizes Ufd1’s UT3 domain. Yet to further validate this hypothesis and our structural data for the ternary complex, we sought out point mutations in Faf1 and Ufd1 that sterically break the binding interface. Indeed, the S518K mutation in Faf1 or the A140E mutation in Ufd1 abolished the accelerated substrate unfolding by p97-UN-Faf1 (Figures 5A and 5B; Figure S15). Ufd1 A140E also reduced the photo-induced crosslinking between ubiquitin and the BPA incorporated at position 501 of Faf1 (Figure S9) but had no effect on substrate unfolding in the absence of Faf1 (Figure 5B).

Faf2 is a membrane-associated cofactor that is involved in ERAD^28,29^ but shows partial conservation with Faf1 (Figure S13). AlphaFold predicts that Faf2’s C-terminal UBX domain interacts with p97’s NTD, while the preceding FH binds to Ufd1’s UT3 domain (Figure 5D). We generated two truncated Faf2 fragments: one lacking the first 271 residues, which leaves the C-terminal helix and UBX domain, and another lacking the first 296 residues, which in addition removes about half of FH’s UT3-binding region and represents a fragment previously used in replisome-disassembly studies (Figures 5D and 5E; Figure S1D). In our Eos-unfolding assays, the addition of Faf2^Δ1-271^ to p97-UN led to a 5-fold stimulated activity (Figure 5F), comparable to the FH-UBX domain fragment of Faf1. In contrast, Faf2^Δ1–296^ has some of the UT3-interacting residues removed and failed to accelerate substrate turnover. In summary, these results suggest that the helix preceding the UBX domain in both Faf1 and Faf2 interacts with the UT3 domain of Ufd1 to stimulate substrate processing by p97-UN, likely by stabilizing Ufd1’s position for more productive initiator-ubiquitin unfolding and insertion into the AAA+ motor.

## DISCUSSION

Here, we show that Faf1 and Faf2 accelerate the early steps in the processing of ubiquitinated substrates by p97-UN. The effects depend on the recruitment of these cofactors through their UBX domain to p97’s NTDs, which positions a conserved helix preceding the UBX domain to interact with the UT3 domain of Ufd1. This interaction appears critical for promoting a productive p97-UN complex that can rapidly insert the initiator ubiquitin into the ATPase motor, likely through stabilizing the position of Ufd1’s UT3 domain relative to Npl4. Our cryo-EM data together with AlphaFold modeling indicate that the UT3 domain binds not only the ubiquitin moiety (Ub^prox^) proximally neighboring the initiator ubiquitin (Ub^ini^) but also the C terminus of Ub^ini^ itself, with the latter interaction apparently dependent on Faf1 bracing the UT3 domain. UT3-domain stabilization by Faf1 may thus facilitate the recruitment of the initiator ubiquitin, its unfolding, and its placement in Npl4’s hydrophobic groove for p97 insertion. In addition to a reasonably well-resolved Faf1 moiety that seems to stably interact with the UT3 domain, we observed density for a second Faf1, whose resolution for the long helix preceding the UBX domain depended on the position of the ubiquitin-bound UT3 domain. The two Faf1 copies bind to neighboring NTDs of p97 that are also occupied by the two SHP motifs of Ufd1, suggesting a cooperativity in these interactions and a possible double-sided stabilization of the UT3 domain by two Faf1 molecules.

In the presence of Faf1, we observed ~5-fold faster kinetics of substrate processing by p97-UN. The extent of this acceleration is thereby limited by the slow unfolding of our Eos model substrate, while ubiquitin unfolding and p97 insertion themselves are accelerated by more than two orders of magnitude. Initiation by p97-UN in the absence of Faf1 is apparently highly inefficient. Unlike Cdc48-UN, which shows maximum initiation and substrate-unfolding rates with ubiquitin chains of six moieties and reasonable activities even with dimeric chains,^14^ p97-UN requires much longer ubiquitin chains for any detectable unfolding activity.^19^ Our finding that Faf1 strongly facilitates ubiquitin engagement thus explains previous reports about Faf1 lowering the chain-length threshold for p97-UN.^19^ Directly bridging p97’s NTD and Ufd1’s UT3 domain through interactions with Faf1’s UBX domain and rigid helix thereby seems essential, and it is conceivable that other UBX-domain-containing proteins use similar multivalent contacts to help coordinate the myriad of p97 cofactors. Interestingly, Faf1 contains several other domains, including a ubiquitin-binding UBA domain^26^ whose truncation did not cause significant defects in our unfolding experiments. Faf1 may thus engage in other substrate-specific interactions^23,27^ or even function in other pathways.^32,40^

Initiation by p97-UN in the presence of Faf1 occurs similarly fast as by Cdc48-UN, yet unfolding the Eos moiety of our model substrate takes ~200-fold longer for p97 than for Cdc48. This is likely caused by the vastly different rates in ATP hydrolysis for these human and yeast orthologs. Previous studies of GFP degradation by the bacterial AAA+ protease ClpXP showed that successful unfolding of the beta-barrel structure critically depends on the pulling frequency of the motor,^41^ such that p97 may have a considerable disadvantage in unraveling Eos and potentially have to compete with the fast refolding of a short-lived unfolding intermediate. Future studies will have to address whether p97 could benefit in its substrate-unfolding velocity from other cofactors that either stimulate ATP-hydrolysis or act as chaperones to destabilize the folded domains of substrate proteins.

### Limitations of the study

Due to the flexibility of the poly-ubiquitin substrate as well as the Npl4, Ufd1, and Faf1 cofactors in complex with p97, there is limited resolution of our cryo-EM maps, especially for the ubiquitin-bound UT3 domain, and the *de novo* building of atomic models was not possible. The maps nicely validate the various AlphaFold models and in combination provide exciting insights into the mechanisms of UN-Faf1-mediated initiation of substrate processing by p97. Yet certain aspects, like the detailed binding mode of the initiator ubiquitin’s C terminus interacting with the beta sheet in Ufd1’s UT3 domain, require further investigation, for instance by X-ray crystallography.

Another limitation of the *in vitro* studies lies in the determination of the Faf1 stoichiometry. Although our cryo-EM maps indicate that two Faf1 molecules bind the p97 hexamer on NTDs that are also occupied by the two SHP motifs of Ufd1, the scenario under physiological conditions remains unclear.

## STAR★METHODS

### EXPERIMENTAL MODEL AND STUDY PARTICIPANT DETAILS

The recombinant expression of all proteins used in this study was performed in *E. coli* BL21 DE3 or *E. coli* Rosetta2 pLysS strains, which were grown in DYT (2x Yeast Extract-Tryptone) medium at 37°C and 18°C.

### METHOD DETAILS

#### Purification of recombinant proteins

Expression, purification, and labeling of p97 and p97^D592AzF^ were carried out as previously described.^20,51^ Briefly, after the recombinant expression of wild-type p97 in *E. coli* BL21 DE3, cells were harvested by centrifugation at 3,500 rcf and resuspended in NiA buffer (50 mM HEPES-KOH, pH 7.6, 500 mM KCl, 20 mM imidazole) supplemented with benzonase nuclease, lysozyme, and protease inhibitors. Cells were lysed by sonication, the lysate was clarified by centrifugation at 27,000 rcf, and the supernatant was loaded onto Ni-NTA resin followed by 10 column volumes (CV) washing with NiA buffer. The protein was eluted with NiB buffer (50 mM HEPES, pH 7.6, 500 mM KCl, 400 mM imidazole) and concentrated before loading on a size-exclusion chromatography column (HiLoad Superdex 200 16/60) that was equilibrated with GF buffer (50 mM HEPES-KOH, pH 7.6 150 mM KCl, 5 mM MgCl_2_, 1 mM DTT). Fractions were analyzed by SDS-PAGE, concentrated, and snap frozen by liquid nitrogen. For p97^D592AzF^ expression, cells were co-transformed with the plasmids coding for p97^D592AzF^ and the AzF-tRNA synthetase, grown at 37°C to an OD_600_ of 0.6, and incubated with AzF at a final concentration of 1 mM. After 30 min, expression was induced with 0.5 mM IPTG and cells were grown at 18°C for 16 h before harvest.

Expression and purification of human UN complex and mutans were carried our as previously described.^20^ Briefly, *E. coli* BL21 DE3 cells with a plasmid coding for Ufd1 or Npl4 were grown separately and resuspended in NiA buffer together before cell lysis. The supernatant after centrifugation was subjected to Ni-NTA purification and UN was further purified by size-exclusion chromatography (HiLoad Superdex 200 16/60). Peak fractions containing the UN complex were pooled and concentrated before snap freezing.

Faf1, Faf2, and their variants were expressed in Rosetta2 pLysS cells as GST fusions with an HRV-3C protease cleavage sites. Cells were lysed, clarified by centrifugation, and loaded on a glutathione resin equilibrated with GF buffer. After washing with 20 CV of GF buffer, GST-HRV3C protease was added to the resin and incubated at 4°C overnight to cleave off the tag. Cleaved target proteins were washed off from the resin with GF buffer supplemented with 5 mM DTT and further purified by size exclusion chromatography (Superdex 200 increase 10/300).

For expression of Faf1 FH-UBX^R501BPA,E527C^, cells were transformed with a plasmid coding for Faf1 FH-UBX^E527C^ carrying an amber stop codon at residue 501 and the vector encoding the BPA aminoacyl-tRNA synthase (MjTyrRS)/tRNA pair for BPA incorporation.^52,53^ Cells were grown at 37°C to an OD_600_ of 0.6, and incubated with pBPA dissolved in NaOH at a final concentration of 2 mM. After 30 min, expression was induced with 0.5 mM IPTG and cells were grown at 18°C for 16 h before harvest.

Wild-type human ubiquitin and its variants were purified as previously reported.^14^ In brief, for wild-type ubiquitin and ubiquitin with an N-terminal Met-Cys extension (MC-Ub), cells were lysed in NiA buffer, the supernatant was clarified by centrifugation at 27,000 rcf for 30 min, and glacial acetic acid was gradually added until the solution reached a pH of ~4.5. Precipitants were separated by centrifugation at 27,000 rcf for 30 min, the supernatant was dialyzed at 4°C for 16 h against 50 mM sodium acetate buffer, pH 4.5, and additional precipitants were removed by centrifugation. The supernatant was loaded to a cation-exchange column (5 mM HiTrap SP HP, Cytiva) equilibrated with 50 mM sodium acetate buffer, pH 4.5, and proteins were eluted with a linear gradient of 1–60% of 1 M NaCl over 30 CV. Peak fractions containing the target protein were pooled and concentrated, and proteins were further purified by size exclusion chromatography (HiLoad Superdex 75 16/60) in GF buffer. For Ub-His and MC-Ub-His, a Ni-NTA purification step was performed instead of the acidic precipitation process. For all MC-Ub variants, 5 mM DTT was added to the GF buffer for the final size exclusion chromatography to keep cysteines reduced.

The plasmid for expression of His-SUMO-Ub^G76V^-mEos3.2 was synthesized by GenScript and used to transform BL21 Star (DE3) competent cells. Expression was induced at OD_600_ = 0.6, and cell were grown at 20°C for 16 h before harvest. After pelleting, cells were resuspended in NiA buffer and lysed by sonication. The lysate was cleared by centrifugation and the supernatant was loaded on Ni-NTA resin pre-equilibrated with NiA buffer. After washing with 20 CV NiA buffer, the target protein was eluted with NiB buffer. Imidazole in the solution was diluted using Amicon spin filters before the addition of SENP2 protease. The cleavage was performed at 4°C for 16 h, and cleaved Eos was isolated as the flow through fraction of a subtractive Ni-NTA purification. Samples were concentrated and further purified by size-exclusion chromatography (HiLoad Superdex 75 16/60).

For the fusion protein of Eos-Turquoise, BL21Star (DE3) competent cells were transformed with pET24 His-3C-Ub^G76V^-mEos3.2-Turqoise and grown as described above. The protein was purified by Ni-NTA affinity in NiA buffer and size-exclusion chromatography in GF buffer with the His tag uncleaved.

Mouse E1 (Ube1)^54^ and the E2-E3 chimera gp78RING-Ube2g2^20^ were expressed and purified as described.

#### Protein labeling with fluorophores

After Ni-NTA affinity purification, p97^D592AzF^ was treated with 5-fold molar excess of DTNB for 30 min at room temperature to protect solvent-exposed cysteines and then labeled with 5-fold molar excess of LD655-DBCO (custom synthesis by Lumidyne Technologies) for 2 h at room temperature. The reaction was quenched with an excess amount of free AzF and treated with 5 mM DTT before a final purification by size exclusion chromatography (HiLoad Superdex 200 16/60) in GF buffer. The efficiency of labeling was estimated based on the protein concentration measured by BCA assay in comparison with LD655 absorbance (typically 20–30%).

MC-Ub and its variants were labeled with Cy3-or Cy5-maleimide dyes at the N-terminal engineered cysteine. First, MC-Ub was treated with 1 mM TCEP for 30 min at room temperature, followed by the addition of 10-fold molar excess of sulfo-Cy3-maleimide or sulfo-Cy5-maleimide (Lumiprobe) dissolved in DMSO. The final concentration of MC-Ub was 500 μM, and the labeling reactions were carried out at room temperature for 30 min. Reactions were quenched by adding DTT to a final concentration of 10 mM, the protein was concentrated and subjected to size-exclusion chromatography (Superdex 75 increase 10/300) in GF buffer. Protein concentration was estimated based on Cy3 or Cy5 absorbance. Faf1 FH-UBX^R501BPA,E527C^ was labeled in a similar way as MC-ubiquitins, since C527 is the only cysteine in this fragment.

#### Preparation of ubiquitinated Eos and Eos-Turquoise substrates

The reaction mixture for ubiquitination consisted of 7.5 μM E1, 125 μM chimeric E2-E3, 100 μM Ub^G76V^-mEos3.2 or Ub^G76V^-mEos3.2-Turquoise, 500 μM ubiquitin and GF buffer with 10 mM ATP. The reaction was carried out in a total volume of 500 μL at 37°C for 2.5 h. An extra 500 μM ubiquitin was added every 30 min to efficiently elongate the ubiquitin chain. The mixture was subsequently subjected to size-exclusion chromatography (Superose 6 increase 10/300), and fractions with substrate-attached ubiquitin chain of more than 10 Ub were pooled and concentrated. To prepare the red Eos, purified ubiquitinated green Eos was photoconverted on ice for 30 min using a 395nm 50W LED Black Light lamp (CONALUX). The conversion efficiency was ~50–60% based on the fluorescence ratio.

#### Assembly of ubiquitin chains

To prepare Ub-^Cy3^Ub-Ub_n_ and ^Cy5^Ub-^Cy3^Ub-Ub_n_ chains used for FRET-based experiments, the first step was to make Ub-His-^Cy3^Ub and ^Cy5^Ub-His-^Cy3^Ub.^16^ Ub-His or ^Cy5^Ub-His was mixed with a limited amount of ^Cy3^Ub in the presence of the ubiquitination machinery in reaction buffer (50 mM HEPES-KOH, pH 7.6, 150 mM KCl, 5 mM MgCl_2_, 0.5 mM DTT and 10 mM ATP) at final concentrations of 1.5 μM Ube1, 25 μM gp78RING-Ube2g2, 250 μM Ub-His or ^Cy5^Ub-His, and 50 μM ^Cy3^Ub. Reactions were incubated at 37°C for 40 min before loading them onto Ni-NTA resin equilibrated with NiA buffer. Proteins were eluted with NiB buffer, concentrated, and subjected to size-exclusion chromatography (Superdex 75 increase 10/300) in GF buffer. Peak fractions were analyzed by SDS-PAGE, and fractions containing di-Ub were pooled and concentrated. Ub-His-^Cy3^Ub or ^Cy5^Ub-His-^Cy3^Ub were elongated with unlabeled ubiquitin in the presence of ubiquitination machinery at 37°C for 2.5 h. Final concentrations were 7.5 μM Ube1, 125 μM gp78RING-Ube2g2, 100 μM Ub-His-^Cy3^Ub or ^Cy5^Ub-His-^Cy3^Ub, and 1 M ubiquitin, with ubiquitin being added in 200 mM aliquots every 30 min. Ubiquitin chains were purified by Ni-NTA affinity and size-exclusion chromatography (Superdex 200 increase 10/300). Fractions with chains containing more than 8 Ub moieties were pooled and concentrated.

#### Single-turnover unfolding assays

Single-turnover unfolding assays for ubiquitinated Eos or the Eos-Turquoise fusion substrate were performed using a BMG Labtech CLARIOstar plate reader at 37°C. Substrate was preincubated with ATP regeneration mix (see below), and p97 was incubated with cofactor(s) in assay buffer (50 mM HEPES-KOH, 150 mM KCl, 5 mM MgCl_2_, 1 mM DTT). The reaction was initiated by mixing the substrate with p97/cofactors in 1:1 volume ratio. The final concentrations were 20 nM substrate, 400 nM p97, 2 μM UN and/or 2 μM Faf, and 1X ATP regeneration mix (5 mM ATP, 0.03 mg/mL creatine kinase, 16 mM creatine phosphate). Unfolding was monitored by measuring the loss of fluorescence, at 585 nm after excitation at 565 nm for red Eos, at 520 nm after excitation at 500 nm for green Eos, and at 475 nm after excitation at 415 nm for Turquoise. Fluorescence traces were fitted by single-exponential decay functions using GraphPad Prism.

#### Complex assembly and cryo-EM sample preparation

##### Sample for dataset 0

p97 was pre-incubated with UN, full-length Faf1, and Eos substrate carrying ubiquitin chains with ~8 Ub moieties at 37°C for 10 min, before addition of 2 mM ATP and 10 more minutes of incubation. The final concentrations were 10 μM p97, 20 μM UN, and 20 μM Faf1. The mixture was subjected to size-exclusion chromatography (Superose 6 increase 10/300) in GF buffer supplemented with 2 mM ATPγS. Peak fractions were concentrated, concentrations were determined with BCA assays, and samples were snap frozen. For grid preparation, 3 μL of 4 mg/mL assembled complex was applied to an UltrAuFoil grid (r1.2/1.3, 300 mesh) that was glow-discharged using PELCO easiGlow system (25 mA, 25 s), and grids were plunge-frozen in liquid ethane using a Vitrobot Mark IV (Thermo Fisher Scientific) set to 4°C and 100% humidity with 4 s blot time.

##### Sample for dataset 1 and 2

^LD655^p97^D592AzF^ was pre-incubated with UN and Faf1 FH-UBX, and Ub-^Cy3^Ub-Ub_n_ was diluted in GF buffer supplemented with 5 mM ATP, 0.06% CHAPS, and 0.04% octyl-β-D-glucopyranoside on ice. p97/UN/Faf1 was then mixed with ubiquitin chains/ATP, and immediately loaded onto a grid (Ultrathin carbon on Quantifoil, R2/2, glow discharged in easiGlow, 25 mA, 90 s), followed by plunge freezing using Vitrobot (4°C, 100% humidity, 2 s wait time, 3 s blot time). The final concentrations were 12.5 μM ^LD655^p97^D592AzF^, 10 μM UN, 10 μM Faf1 FH-UBX, and 20 μM Ub-^Cy3^Ub-Ub_n_.

#### Cryo-EM data collection, single-particle analysis, and model building

Grids were screened on a 200 kV Talos Arctica microscope (Cal Cryo Facility at UC Berkeley) and data collection was performed at the HHMI Janelia Research Campus, using SerialEM^55^ on a 300 kV Titan Krios G4 equipped with SelectrisX energy filter (6eV slit width) and a Falcon4i camera. For dataset 0, 24,016 movies were recorded at a nominal magnification of ×165,000 (0.743 Å/pix) with a total dose of 50 e^−^ /Å^2^ nd a defocus range of −0.8 to −1.6 μm. For dataset 1 and 2, 10,496 and 6,748 movies were recorded at a nominal magnification of ×105,000 (1.182 Å /pix) with a total dose of 50 e^−^ /Å^2^ and a defocus range of −0.8 to −1.6 μm. Detailed data processing steps are shown in Figures S3 and S5.

For datasets 0, multi-frame movies were imported into cryoSPARC Live for motion correction and CTF estimation, and downstream steps were carried out using cryoSPARC v4.6.^49,56^ Particles picked with Blob picker were subjected to 2D classification and ab-initio reconstruction to get rid of junk particles. A subset of the particle stack was used as seeds for Topaz training, and the trained model was used to extract particles out of the whole dataset.^57,58^ After extraction, the particles were subjected to 2D classification, ab-initio reconstruction, and heterogeneous refinement. The main class with p97 NTD in the “up” conformation was further refined with C6 symmetry, and the density of five p97 subunits was subtracted after symmetry expansion to leave out a single subunit for focused 3D classification and local refinement of the p97-NTD/Faf1-UBX region.

For dataset 1 and 2, images were pre-processed as those in dataset 0. After initial hetero-refinement, particles in the major class were subjected to 3D classification with a mask covering the region above the D2 domain of p97. Two classes with clear Npl4 density were chosen for further particle sorting. For the one with tilted Npl4 density (PI state), further classification did not improve the heterogeneity on top of the Npl4 tower, likely due to Npl4’s flexibility in this state, and we therefore performed a global refinement. For the other state with more stable Npl4 density (I state), we first aligned particles on the p97 motor (motor focused map), and noticed heterogeneity on top of Npl4. This motivated us to align particles on Npl4, followed by 3D classification with a Npl4-focused mask. One class showed clear density for three distal ubiquitins and was further locally refined as Npl4 local map. Several other classes showed potential heterogeneity around the first distal ubiquitin, Ub^dist1^, but the numbers of particles were not sufficient for further processing. Another unique feature in the I state map is the Ufd1 UT3 density between two Faf1 helices. We performed 3D classification with a focused mask covering Faf1 FH-UBX and UT3, and chose two of these classes for further global refinement (IC1 and IC2).

For model building, an Alphafold3-predicted structural model was docked into the local map, manually adjusted using ISOLDE^46^ in ChimeraX,^59^ COOT,^60^ and finally refined using PHENIX real space refinement.^61^

#### ATPase assays

The rate of ATP hydrolysis was measured using a coupled assay that monitors the decrease in NADH absorbance^62^ in a plate reader at 37°C. p97/cofactors and the substrate/ATPase mix (5 mM ATP, 1 mM NADH, 7.5 mM phosphoenolpyruvate, 12 U/mL pyruvate kinase, 18 U/mL lactate dehydrogenase) were pre-incubated separately and mixed in 1:1 volume ratio right before the measurement. The absorbance of NADH at 340 nm was monitored, and ATPase rates were determined by linear regression. The final concentrations were 50 nM p97, 250 nM UN, 250 nM Faf variant, and 250 nM ubiquitinated Eos substrate.

#### FRET-based pore-insertion and initiator-unfolding assays

Steady-state FRET assays were performed using a BMG Labtech CLARIOstar plate reader at 37°C. p97 was pre-incubated with UN/Faf1 in assay buffer, and labeled polyubiquitin chains were incubated separately in assay buffer supplemented with ATP or ATPγS for 10 min p97/UN/Faf1 was mixed with substrate in a 1:1 volume ratio and transferred to a 384-well plate. The plate was incubated in the plate reader at 37°C for 1 min before scanning the fluorescence emission from 550 nm to 750 nm after excitation at 480 nm. The final concentrations were 200 nM ^LD655^p97, 2 μM UN, 2 μM Faf variant, and 200 nM Ub-^Cy3^Ub-Ub_n_ for pore insertion assays. For the initiator-unfolding assay, final concentrations were 2 μM p97, 2 μM UN, 2 μM Faf variant, and 200 nM ^Cy5^Ub-^Cy3^Ub-Ub_n_. The fluorescence intensities for each spectrum were normalized relative to the Cy3 emission maximum at 568 nm.

The kinetics of initiator insertion into p97 were measured in an Auto SF120 stopped-flow fluorometer (Kintek). The syringes were loaded with 145 μL of ^LD655^p97/UN/Faf1 and substrates/ATP, respectively, and incubated at 37°C. After loading, 7 shots of 35 μL each were fired, and the last three were analyzed. The final concentrations were 1 μM ^LD655^p97/UN/Faf1 and 0.1 μM Ub-^Cy3^Ub-Ub_n_ chains. An excitation wavelength of 480 nm was used, and the fluorescence of donor and acceptor dyes were measured simultaneously in different channels.

#### Gel-based crosslinking assay

Purified components were diluted and mixed in GF buffer supplemented with 2 mM ATP on ice, followed by a UV exposure with a 365 nm UV lamp for 45 min. The final concentrations were 2 μM p97, 4 μM UN or U^A140E^N, 2 μM Faf1 FH-UBX^R501BPA,E527C^ and/or 4 μM Ub-^Cy3^Ub-Ub_n_. Samples after crosslinking were analyzed by SDS-PAGE and imaged for Cy5 and Cy3 fluorescence, before Coomassie staining.

#### AlphaFold predictions

All AlphaFold predictions were carried out on the AlphaFold 3 server.^43^ For p97-UN in complex with Faf1 and Faf2, six copies of p97 NTD-D1 fragment (residues 1–451) were used instead of full length p97 due to the token limit. For Ufd1’s UT3 domain in complex with ubiquitins and Faf1 FH, residues 20–192 of Ufd1 and 473–565 of Faf1 were used.

### QUANTIFICATION AND STATISTICAL ANALYSIS

Data were analyzed and fit with Prism 10 (GraphPad Software). Bar graphs in figures show mean values and the standard deviations of the mean, with the number of technical replicates n provided in the figure legends. Statistical significance was calculated using a one-way ANOVA test.

## Supplementary Material

1

Supplemental information can be found online at https://doi.org/10.1016/j.celrep.2026.117393.

## Figures and Tables

**Table:** KEY RESOURCES TABLE

REAGENT or RESOURCE	SOURCE	IDENTIFIER
Bacterial and virus strains		
*E. coli* BL21 Star (DE3)	QB3 MacroLab, UC Berkeley	N/A
*E. coli* Rosetta (DE3) PLysS	QB3 MacroLab, UC Berkeley	N/A
Chemicals, peptides, and recombinant proteins		
Sulfo-Cyanine3 maleimide	Lumiprobe	CAT#21380
Sulfo-Cyanine5 maleimide	Lumiprobe	CAT#23380
LD655-DBCO	Lumidyne	N/A
4-Azido-L-phenylalanine HCl	Acrotein ChemBio Inc.	CAT#A-7137
4-Benzoyl-L-phenylalanine	Amatek	CAT#A-0067
CHAPS	Anatrace	CAT #C316
n-Octyl-β-D-Glucopyranoside	Anatrace	CAT #O311
Deposited data		
Cryo-EM map of p97-UN-Faf1^FL^ (locally refined on p97NTD-Faf1UBX-Ufd1SHP)	This paper	EMDB: EMD-73536
Molecular model of p97-UN-Faf1^FL^ (locally refined on p97NTD-Faf1UBX)	This paper	PDB: 9YW2
Cryo-EM map of p97-UN-Faf1FH-UBX (PI state)	This paper	EMDB: EMD-76074
Molecular model of of p97-UN-Faf1FH-UBX (PI state)	This paper	PDB: 11VE
Cryo-EM map of p97-UN-Faf1FH-UBX (I state)	This paper	EMDB: EMD-76028
Molecular model of of p97-UN-Faf1FH-UBX (I state)	This paper	PDB: 11TA
Cryo-EM map of p97-UN-Faf1FH-UBX (Npl4 local)	This paper	EMDB: EMD-76026
Molecular model of p97-UN-Faf1FH-UBX (Npl4 local)	This paper	PDB: 11SY
Cryo-EM map of p97-UN-Faf1FH-UBX (IC1)	This paper	EMDB: EMD-76053
Cryo-EM map of p97-UN-Faf1FH-UBX (IC2)	This paper	EMDB: EMD-76054
Recombinant DNA		
pET24b-p97-His_6_	Xue et al.^42^	pAM217
pET24b-p97^D592AzF^-His_6_	This paper	pZL1
pET24b-p97^I590AzF^-His_6_	This paper	pZL2
pET24b-p97^N589AzF^-His_6_	This paper	pZL3
pCOLADuet-MGGG-NPLOC4	Blythe et al.^18^	pAM226
pET28a-MSS-UFD1L-His_6_	This paper	pZL14
pET28a-MSS-UFD1L^A140E^-His_6_	This paper	pZL15
pGEX-3C-Faf1	This paper	pZL4
pGEX-3C-Faf1^FH–UBX^	This paper	pZL6
pGEX-3C-Faf1^ΔN^	This paper	pZL11
pGEX-3C-Faf1^ΔUBA^	This paper	pZL12
pGEX-3C-Faf1^ΔUBX^	This paper	pZL13
pGEX-3C-Faf1^UBX^	This paper	pZL5
pGEX-3C-Faf2^Δ1-271^	This paper	pZL19
pGEX-3C-Faf2^Δ1-296^	This paper	pZL20
AzF tRNA synthetase	Williams et al.^14^	pAM293
pET24b-Ub	This paper	pZL21
pET24b-Ub-3C-His_6_	This paper	pZL24
pET24b-MC-Ub	This paper	pZL22
pET24b-MC-Ub-3C-His_6_	This paper	pZL23
His_6_-SUMO-Ub^G76V^-mEos3.2-mTurquoise2-intein-CBD	This paper	pZL25
His_6_-SUMO-Ub^G76V^-mEos3.2	Gift from Nitin Daniel	pAM433
Yeast UFD1-3C-His_6_	Olszewski et al.^30^	pAM294
Yeast NPL4	Olszewski et al.^30^	pAM287
Mouse Ube1	Gift from Jorge Eduardo Azevedo	Addgene plasmid # 32534; RRID:Addgene_32534
His_6_-TEV-gp78RING-Ube2g2	Blythe et al.^18^	pAM304
Software and algorithms		
Prism 10	GraphPad Software	RRID:SCR_002798
AlphaFold3 server	Abramson et al.^43^	RRID:SCR_025885
UCSF Chimera v1.18	Pettersen et al.^44^	RRID:SCR_004097
UCSF ChimeraX v1.10	Pettersen et al.^45^	RRID:SCR_015872
ISOLDE	Croll^46^	RRID:SCR_025577
Coot v0.9.8	Emsley and Cowtan^47^	RRID:SCR_014222
Phenix v2.0	Liebschner et al.^48^	RRID:SCR_014224
CryoSPARC v5.0	Punjani et al.^49^	RRID:SCR_016501
SerialEM	Mastronarde^50^	RRID:SCR_017293

## Highlights

- p97 with its cofactor Ufd1/Npl4 is inefficient in initiating substrate processing
- Faf1 braces Ufd1’s UT3 domain for initiator-ubiquitin unfolding
- The Faf1-UT3 interaction promotes initiator-ubiquitin engagement by p97
