# Supplementary material for: Faf1 accelerates p97-mediated protein unfolding by promoting ubiquitin engagement
Source: Cell Rep. Author manuscript; Available in PMC 2026 Jul 12. (PMC13356908; doi:10.1016/j.celrep.2026.117393)
Supplement: 1 [file NIHMS2190716-supplement-1.pdf]

Cell Reports, Volume *45*

Supplemental information

Faf1 accelerates p97-mediated protein unfolding by promoting ubiquitin engagement

Zengwei Liao, Connor Arkinson, and Andreas Martin

Supplemental Figures

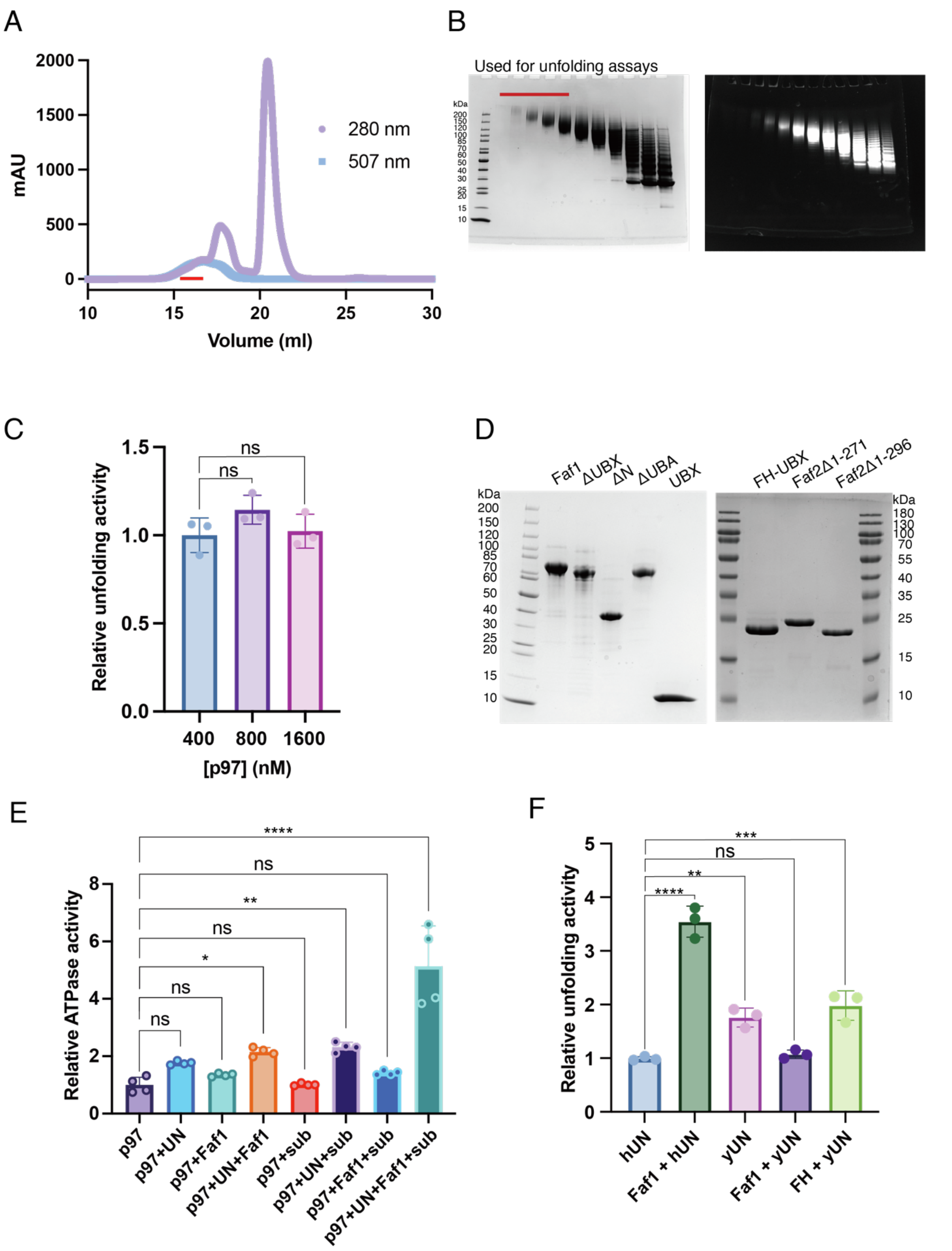

Supplemental Figure 1: Sample preparations for biochemical experiments. A) Size-exclusion chromatogram for the purification of poly-ubiquitinated mEos substrate, monitored

by general protein absorbance at 280 nm (purple) and mEos absorbance at 507 nm (light blue). Fractions pooled and used for unfolding experiments are indicated by a red bar. **B)** SDS-PAGE gels with the peak fractions of the size-exclusion chromatography shown in (A) and visualized by Coomassie staining (left) or mEos fluorescence (right). Fractions pooled and used for unfolding experiments are indicated by a red bar. **C)** Confirmation of single-turnover conditions for substrate unfolding. The rates for unfolding of the Eos substrate (20 nM) in the presence of UN (2 μM) and Faf1 (2 μM) did not significantly change when the p97 concentration was increased from 400 nM to 1600 nM, confirming saturating conditions for single-turnover kinetics. Shown are the mean values of the relative rates and the standard deviation of the mean for 3 technical replicates. Statistical significance was calculated using a one-way ANOVA test: ns, $p>0.05$. **D)** Coomassie-stained SDS-PAGE gels of purified full-length and truncated variants of Faf1 (left) and Faf2 (right). **E)** Relative ATPase activities of p97 in the absence (normalized to 1) and presence of UN, full-length Faf1, and ubiquitinated green Eos substrate. Shown are the mean values and standard deviations of the mean for four technical replicates. Statistical significance was calculated using a one-way ANOVA test: ****$p<0.0001$; **$p<0.01$; *$p<0.05$; ns, $p>0.05$. **F)** Relative rates for the unfolding of poly-ubiquitinated mEos substrate by p97 in the presence of human UN (hUN), yeast UN (yUN), and full-length Faf1 or the FH-UBX fragment. Shown are the mean values and standard deviations of the mean for three technical replicates. Statistical significance was calculated using a one-way ANOVA test: ****$p<0.0001$; ***$p<0.001$;**$p<0.01$; ns, $p>0.05$. The Eos substrate for these series of measurements carried slightly shorter ubiquitin chains (< 8 moieties) than the substrate for the measurements in Fig. 1F (8-12 ubiquitin moieties).

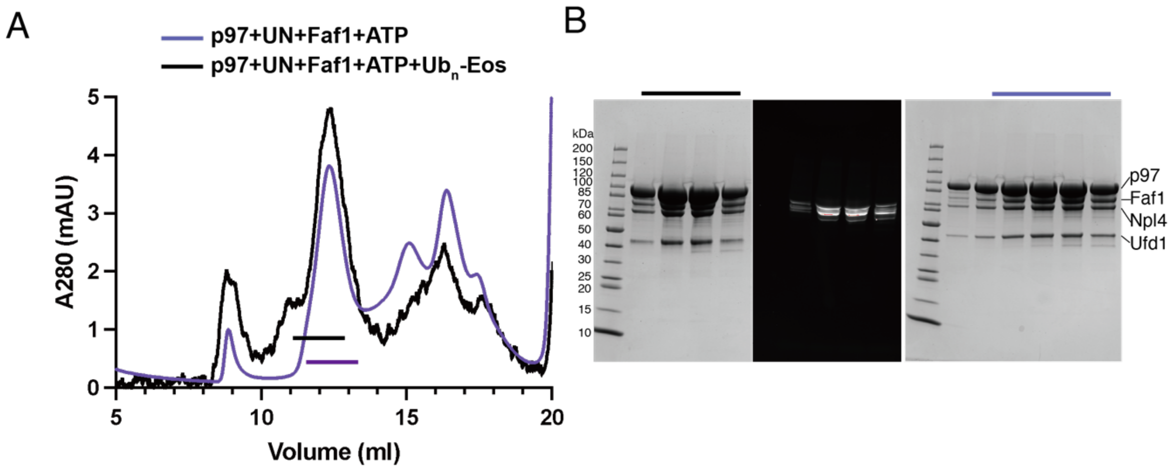

Supplemental Figure 2: Sample preparation for cryo-EM structure determination of the p97-UN complex with full-length Faf1. A) Size-exclusion chromatogram for the purification of the p97-UN-Faf1 complex with ATP in the absence (purple) or presence (black) of poly-ubiquitinated (5-7 moieties) mEos substrate. Horizontal bars indicate fractions that were pooled and used for cryo-EM. **B)** SDS-PAGE gels with the peak fractions of the size-exclusion purifications shown in (A), visualized by Coomassie-staining (left) and mEos fluorescence (middle) for the substrate-containing sample, and visualized by Coomassie-staining for the substrate-free sample (right). Black and purple bars indicate the fractions that contained all components, were pooled, and used for cryo-EM sample preparations.

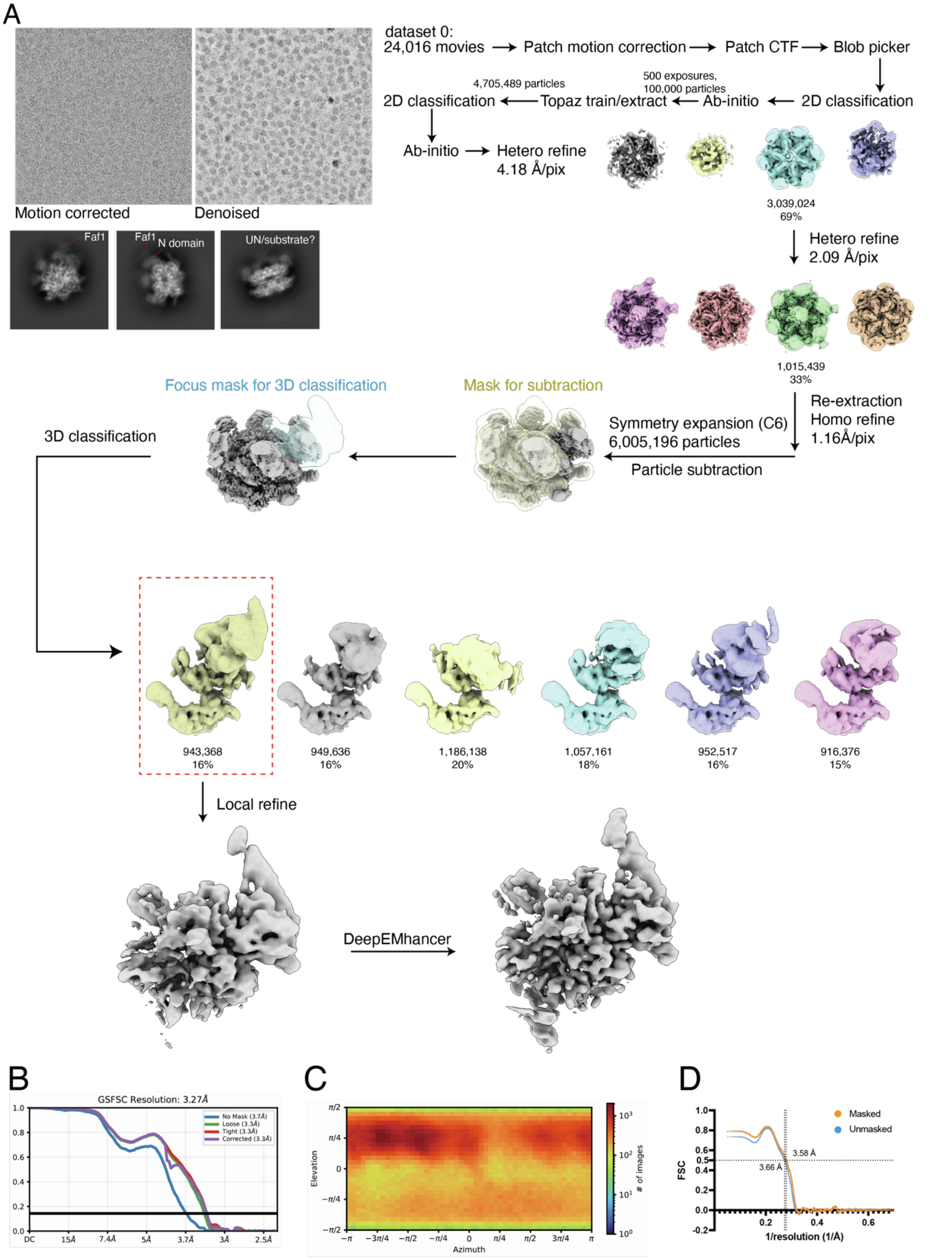

Supplemental Figure 3: Cryo-EM single-particle analysis of p97-UN-Faf1 in complex with a ubiquitinated mEos-model substrate (dataset 0). A) Data-processing workflow using CryoSPARC. **B)** GSFSC curve and **C)** orientation distribution of the final map. **D)** Map-model FSC curve calculated after the final refinement in PHENIX.

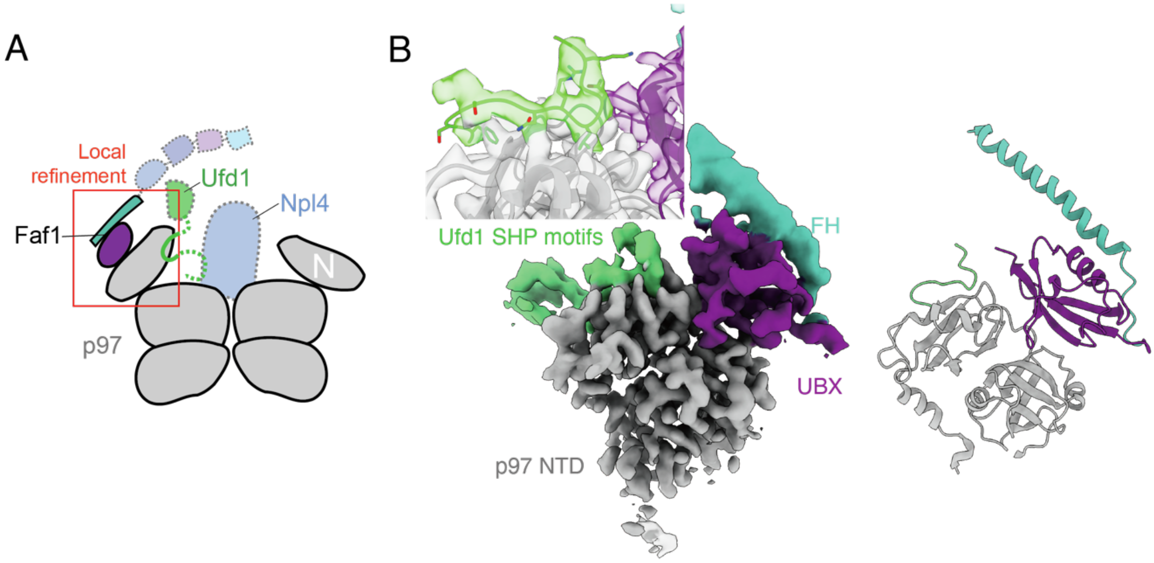

Supplemental Figure 4: Cryo-EM structure determination of the p97-UN complex with full-length Faf1. A) Schematic of the p97-UN-Faf1 complex, with parts not resolved in this cryo-EM structure shown faded and with dashed outlines. The red box indicates the area for focused classification and local refinement in our single-particle analysis. **B)** Cryo-EM density (left) and atomic model (right) of the complex between p97 NTD (grey), Faf1's helix (FH, turquoise) and UBX domain (purple), and Ufd1's SHP motif (green). A focused view of the transparent map and atomic model of the SHP-interacting region is shown on the top left.

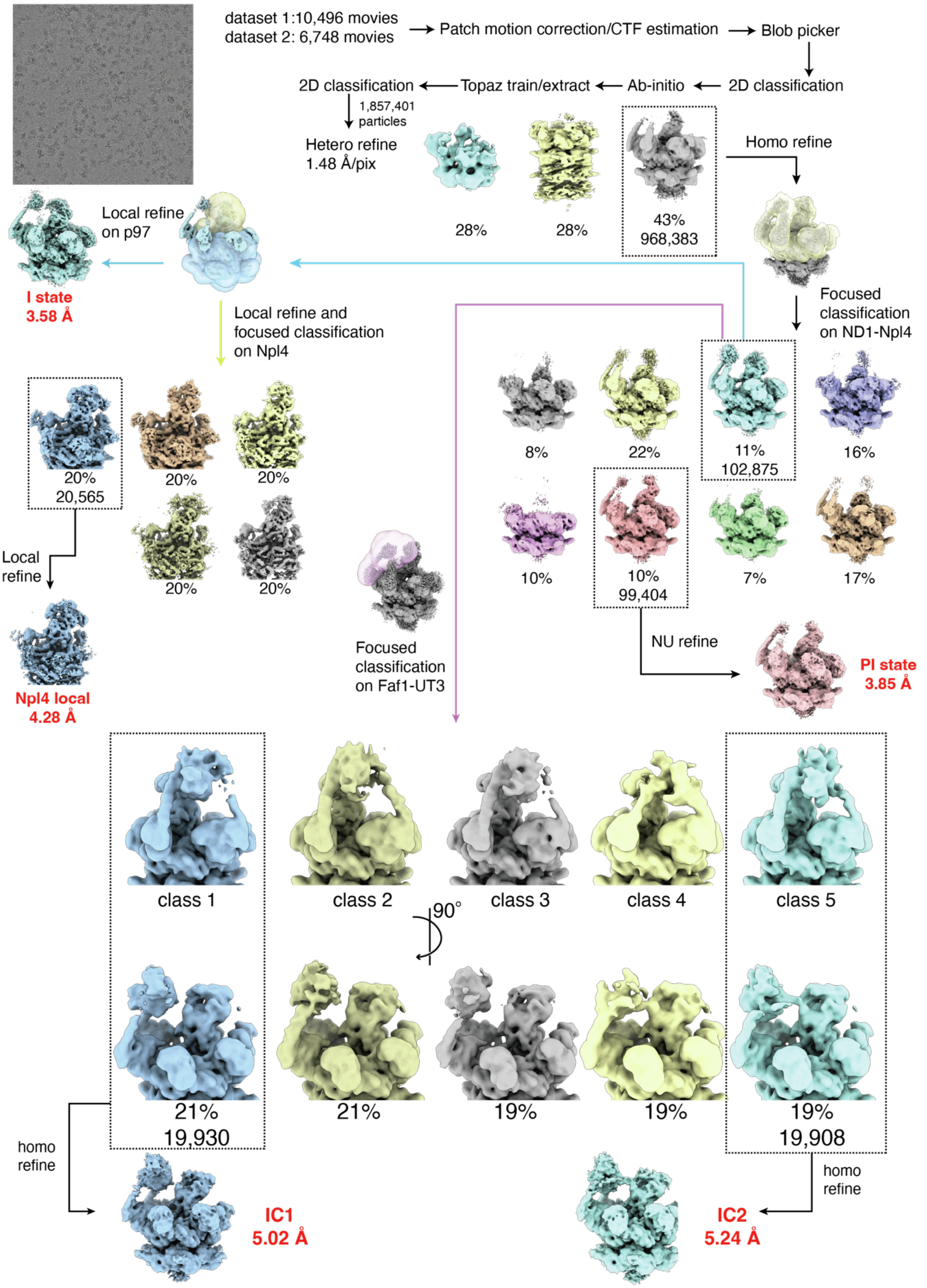

Supplemental Figure 5: Cryo-EM single-particle analysis of p97-UN-Faf1$^{FH\text{-}UBX}$ in complex with unanchored ubiquitin chains complex (dataset 1 and 2).

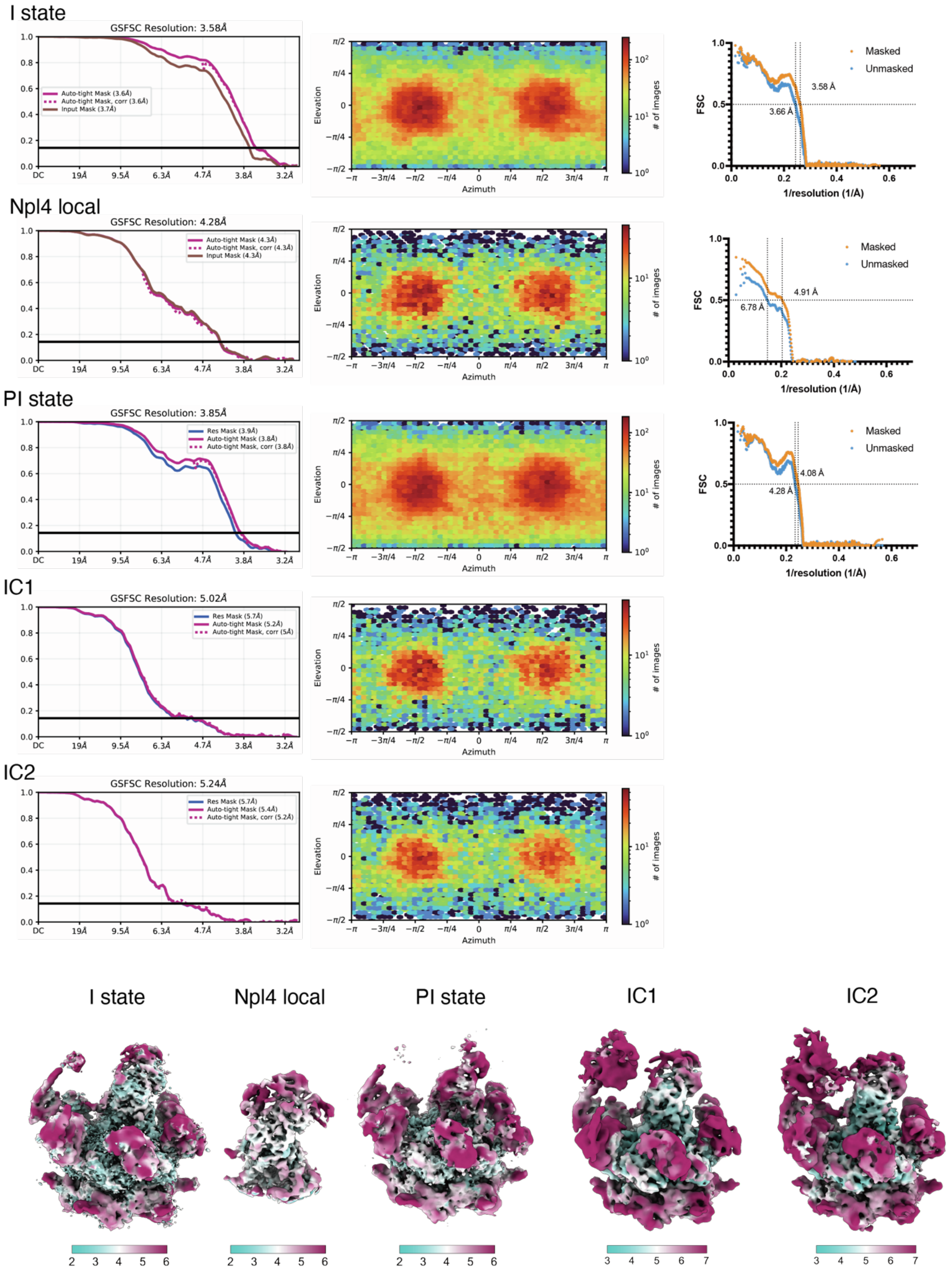

Supplemental Figure 6: FSC curves, particle orientation distributions and local resolution maps for ubiquitin-chain-bound p97-UN-Faf1$^{FH\text{-}UBX}$ ubiquitin bound complexes. The gold-standard Fourier shell correlation (GSFSC) curves are shown for each conformational state, reporting overall resolution at FSC = 0.143. Map-to-model curves are

plotted for the PI state, I state, and the Npl4 local map. Local resolution maps are colored from high resolution (magenta/pink) to low resolution (cyan/teal) as indicated by the color bar beneath each map (Å).

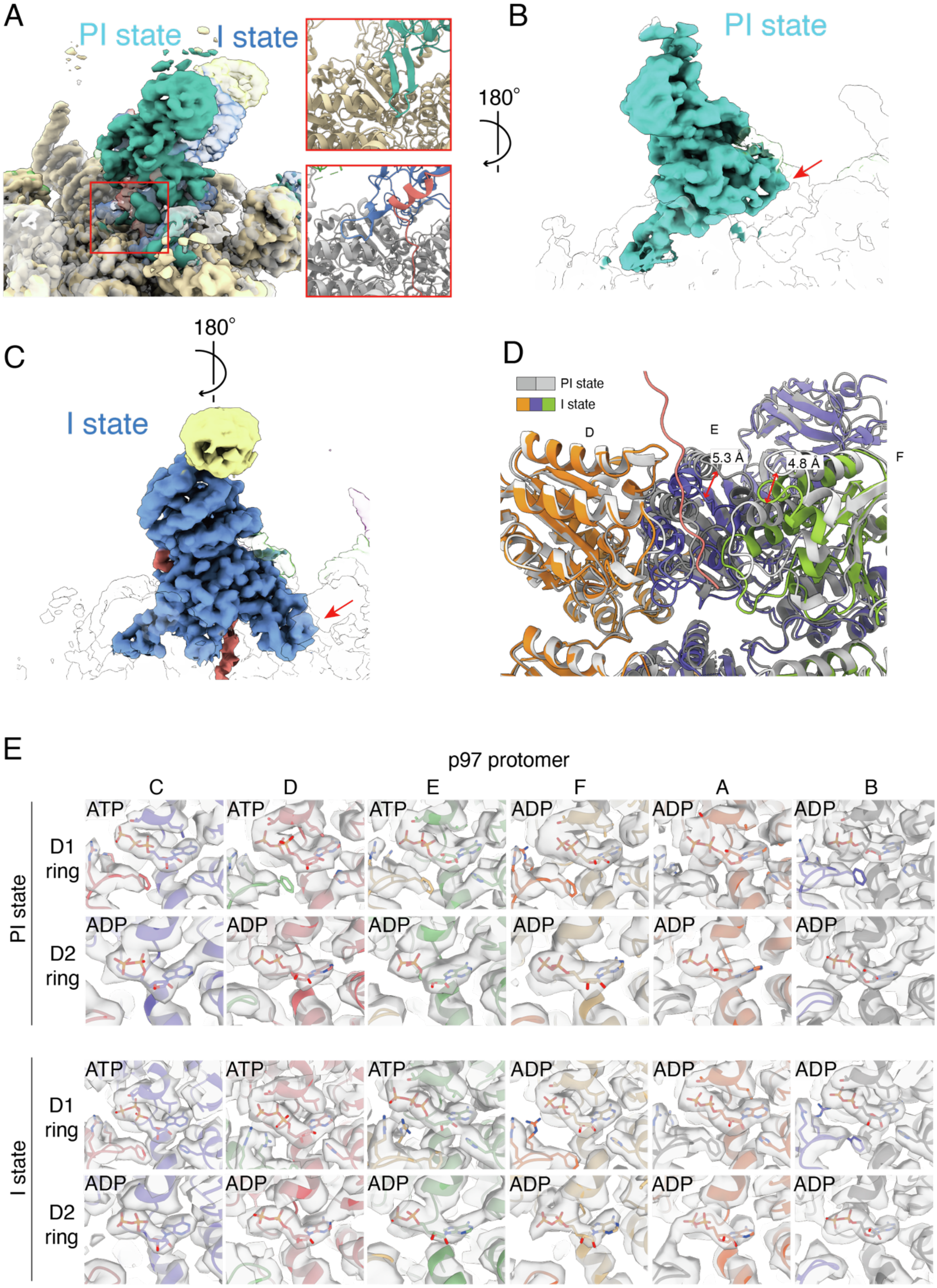

Supplemental Figure 7: Comparisons of pre-initiation (PI) and initiation (I) states of the p97-UN-Faf$^{FH\text{-}UBX}$ in complex with ubiquitin chains. A) Left: Overlay of the cryo-EM densities for the PI state (teal) and I state (blue), illustrating the conformational difference for

Npl4. Right: Insets show zoomed views for the Npl4 loop (residues 425-439) that is positioned above p97's processing channel in the PI state (top box), but relocated to allow insertion of Ub^{ini} (salmon) into p97 in the I state (bottom box). **B,C)** Density map of Npl4 in the PI state (B) and I state (C) is shown from a different angle to highlight the varied conformations of ZF2, indicated with red arrows. **D)** Overlay of the p97 D1 ATPase ring for the PI state (gray) and I state (colored) shows a conformational change of protomers E and F, which in the context of substrate engagement move downward toward the D2 ring. **E)** Cryo-EM densities and atomic models for individual nucleotide-binding pockets of all D1 and D2 ATPase domains in the PI and I states. Each panel is labelled with the identity of the bound nucleotide (ATP or ADP) that is shown in stick representation.

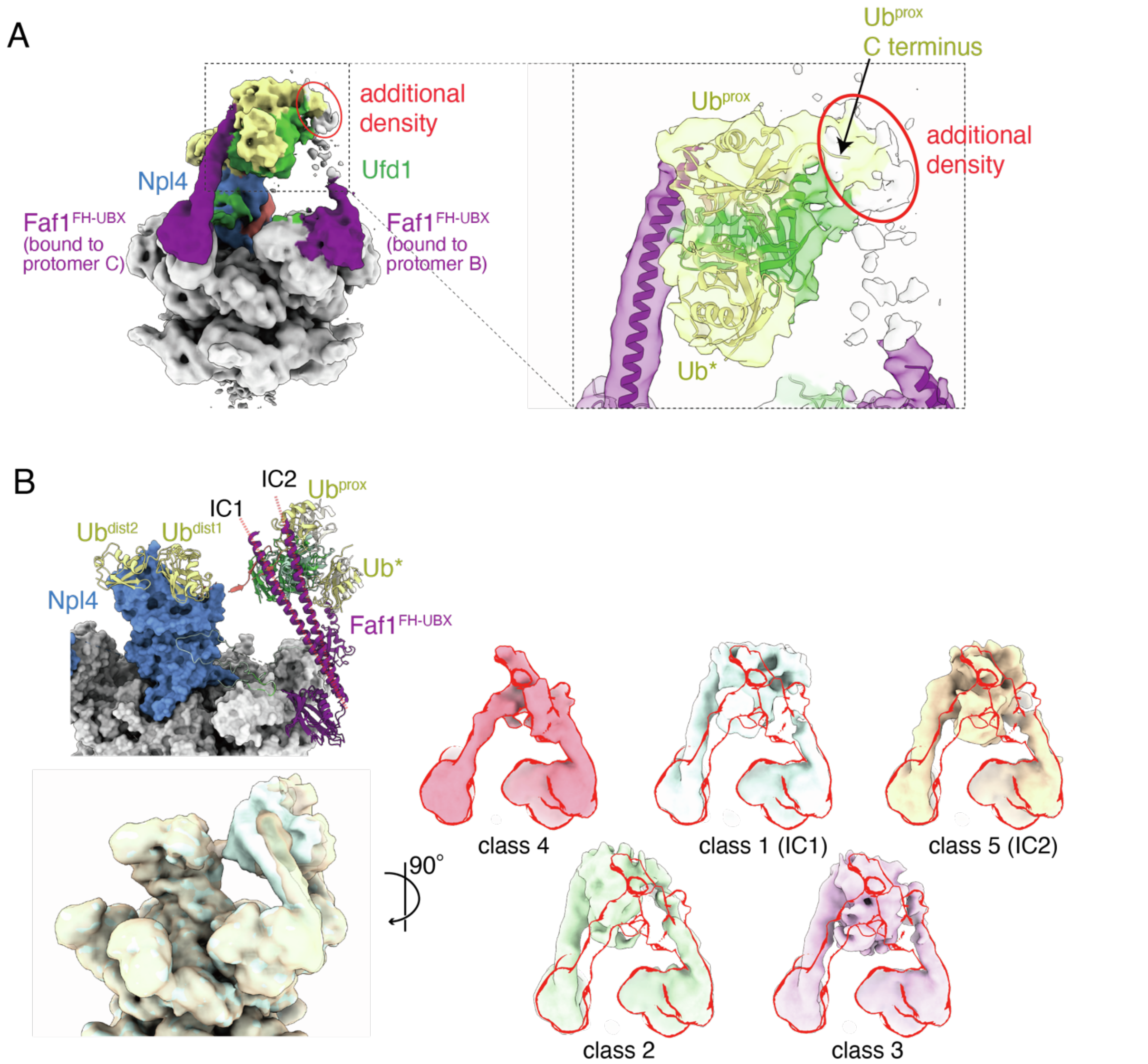

Supplemental Figure 8: Ufd1's UT3 domain is braced by a second Faf1 in a conformation-dependent manner. A) Side view of the cryo-EM density map for the IC1 state (left) and a closed-up view of the UT3-domain region with the docked-in atomic model for the ubiquitin-bound UT3 domain and Faf1 helices (right). **B)** Top left: Models for the IC1 and IC2 states are shown superimposed on the p97-Npl4 region (surface representation), revealing a movement of the ubiquitin-bound UT3 domain (dark colors for IC1, light colors for IC2) and the attached helix of the protomer-C-bound Faf1 away from Npl4 in IC2. Bottom left: Superimposed cryo-EM maps for the 3D classes of IC1 (cyan) and IC2 (yellow) particles. Right: Side views of the Faf1 FH-UT3 regions for five different classes overlayed with class 4 and in the same orientation as in panel A. These overlays highlight the cooperative motion of the UT3 domain and the two FH helices, with variable resolution for the second, protomer-B-bound Faf1.

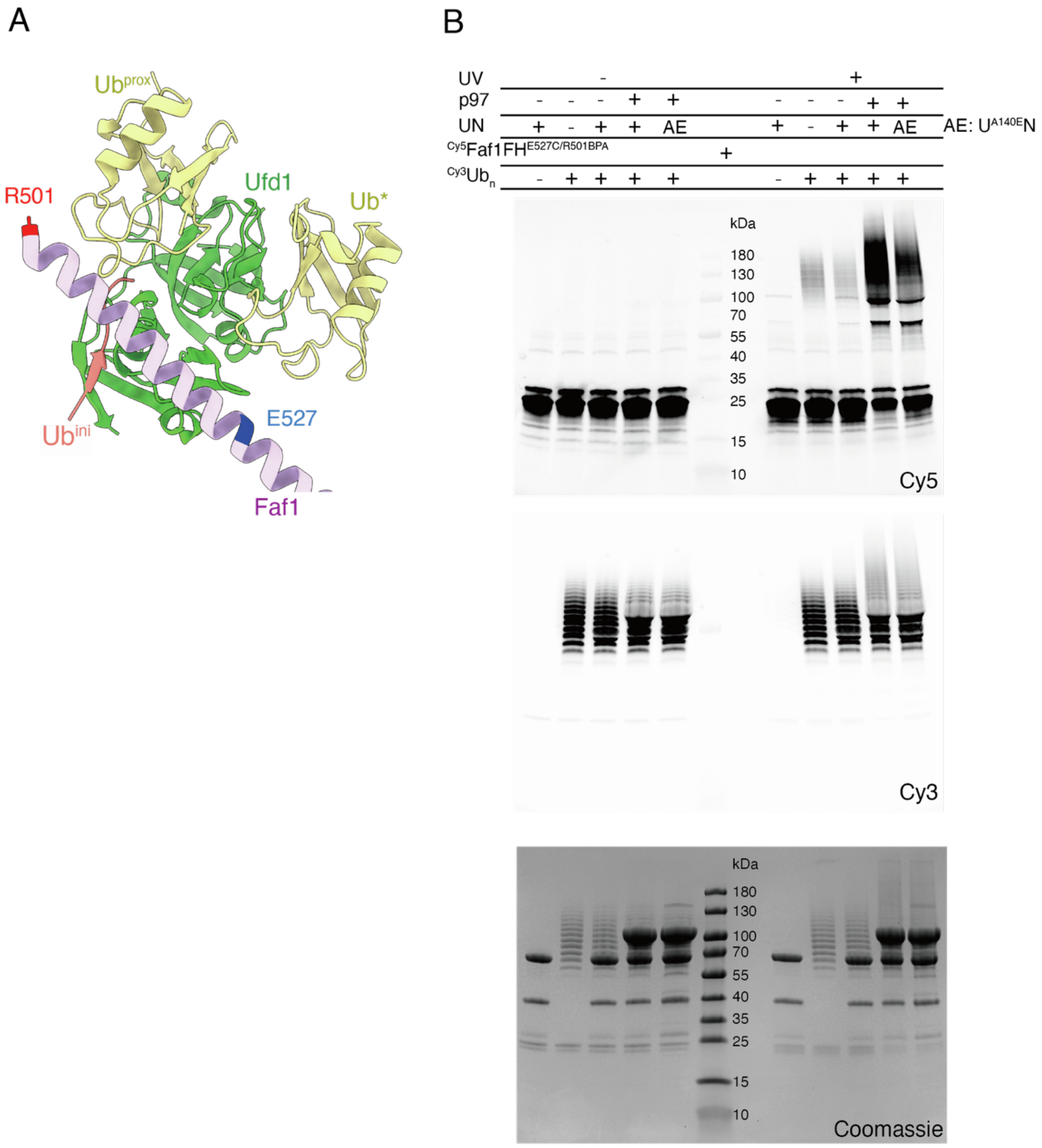

Supplemental Figure 9: Gel-based assay for the photo-induced crosslinking between Faf1 FH and ubiquitin. A) IC1-based atomic model for the UT3-FH-ubiquitin complex to indicate the positions of the E527C mutation for Cy5 labeling and R501BPA incorporation for UV-induced crosslinking of Faf1 FH. **B)** SDS-PAGE gel images scanned for Cy5 (top) and Cy3 fluorescence (middle), and Coomassie-stained (bottom).

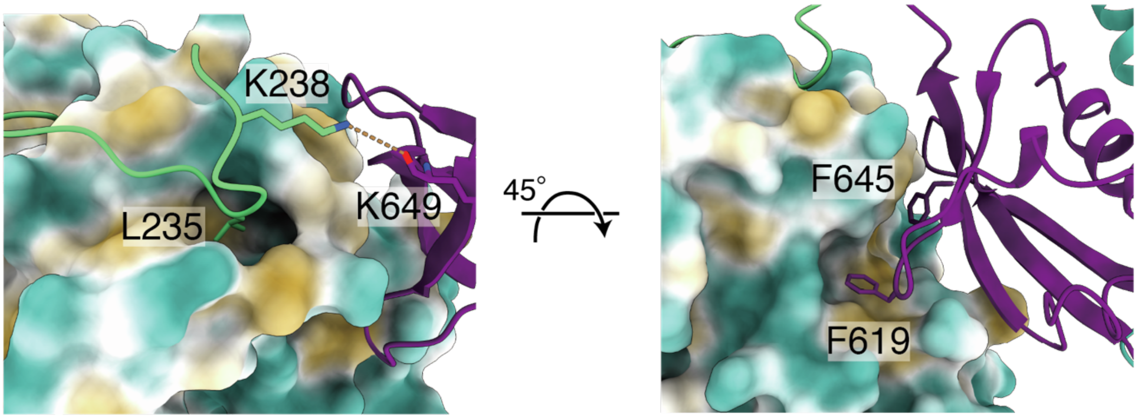

Supplemental Figure 10: Close-up view of interactions between p97 NTD (represented as surface and colored by hydrophobicity), Ufd1's SHP motif (green), and Faf1's UBX domain (purple).

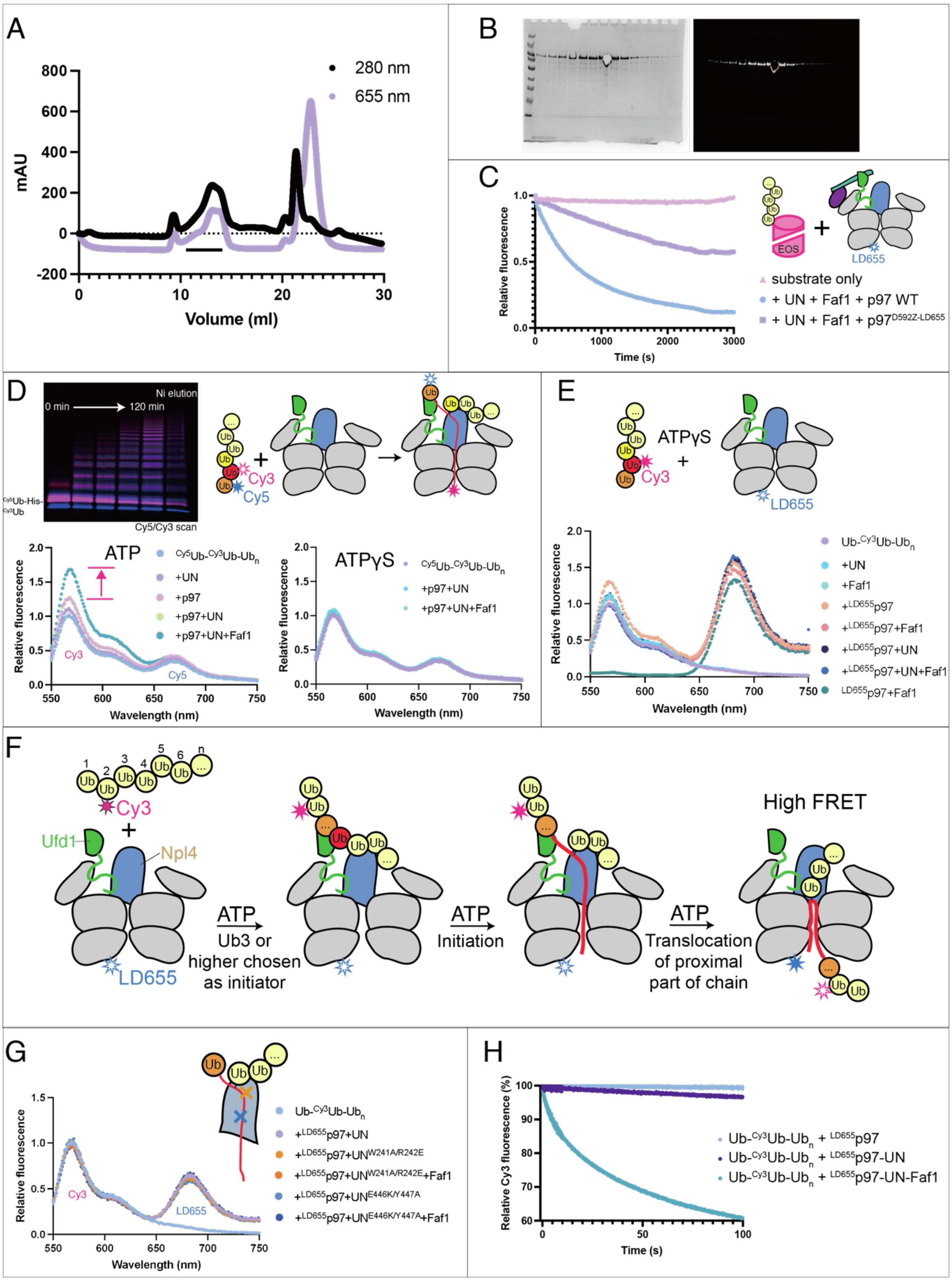

Supplemental Figure 11: Extended results for FRET-based ubiquitin unfolding and initiation assays. A) Size -exclusion chromatogram for the purification of LD655-labeled p97^{D592AzF}, detected by protein absorbance at 280 nm (black) or LD655 absorbance at 655 nm (purple). **B)** SDS-PAGE gels with the peak fractions of the purification show in (A) and

visualized by Coomassie stain (left) or LD655-fluorescence scan (right). **C)** Example traces for the mEos-substrate unfolding by wild-type p97 or LD655-labeled p97, both in the presence of UN and Faf1 cofactors. **D)** Initiator-ubiquitin unfolding measured by the loss of FRET between Cy5 attached to the first ubiquitin and Cy3 attached to the second ubiquitin in unanchored ubiquitin chains (^{Cy5}Ub-^{Cy3}Ub-Ub$_n$) after incubation with p97, UN, and Faf1. The gel image on the top left shows an overlay of Cy3- and Cy5-fluorescence scans for the SDS-PAGE analysis of doubly labeled ^{Cy5}Ub-^{Cy3}Ub-Ub$_n$ synthesis progression over 120 min and the final sample after Ni-NTA affinity purification. Shown at the bottom are the normalized fluorescence emission spectra after excitation at 480 nm for the samples incubated with ATP (left) or ATPγS (right). **E)** FRET-based unfolding initiation assay with unanchored Ub-^{Cy3}Ub-Ub$_n$ ubiquitin chains, ^{LD655}p97, UN, and Faf1 as shown in Fig. 4, but using ATPγS instead of ATP. Shown are normalized example spectra for the fluorescence emission after excitation at 480 nm. **F)** FRET-based initiation assay with unanchored Ub-^{Cy3}Ub-Ub$_n$ ubiquitin chains and ^{LD655}p97 as shown in Fig.4A, but assuming that UN chooses ubiquitin 3 or higher in the chain as the initiator moiety, such that ATP-dependent unfolding and translocation of the proximally located ubiquitins is required to move the Cy3-labeled second ubiquitin into proximity of LD655 for a FRET-signal change. **G)** Same FRET-based initiation assay, but using Npl4 variants carrying W241A/R242E (orange) or E446K/Y447A double mutations (blue) in the hydrophobic groove that prevent capturing the unfolded initiator ubiquitin. Shown are the normalized fluorescence emission spectra after Cy3 excitation at 480 nm. **H)** Effects of Faf1 on the kinetics of unfolding initiation by p97-UN. Shown are example traces for the Cy3-fluorescence quenching after stopped-flow mixing of unanchored Ub-^{Cy3}Ub-Ub$_n$ ubiquitin chains with ^{LD655}p97-UN in the absence or presence of Faf1.

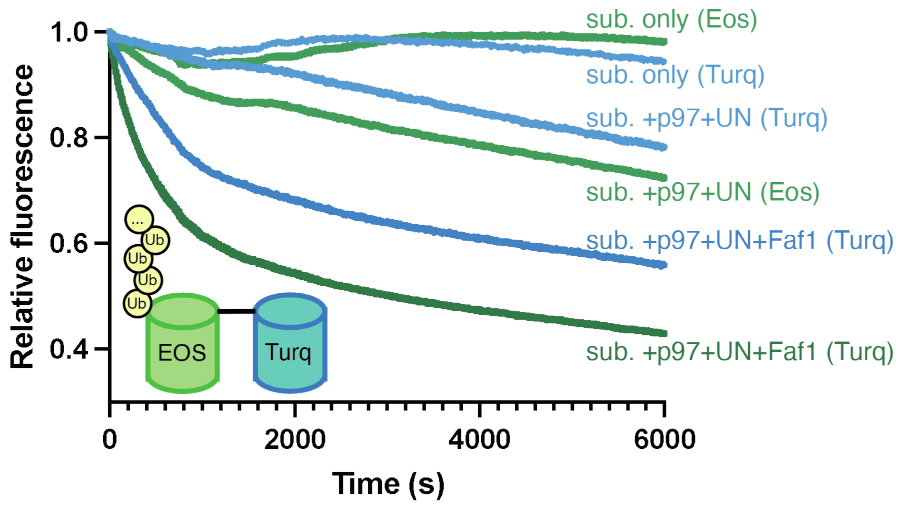

Supplemental Figure 12: Kinetics for the unfolding of the poly-ubiquitinated mEos-Turquoise fusion substrate (schematic inserted in the bottom left) by p97-UN in the absence and presence of Faf1. Shown are example traces for the changes in green Eos fluorescence (shades of green) and changes in Turquoise fluorescence (shades of blue) that were measured separately after mixing the fusion substrate with p97 and cofactors. Loss in Turquoise fluorescence indicates that the Eos moiety of the substrate was completely unfolded and translocated.

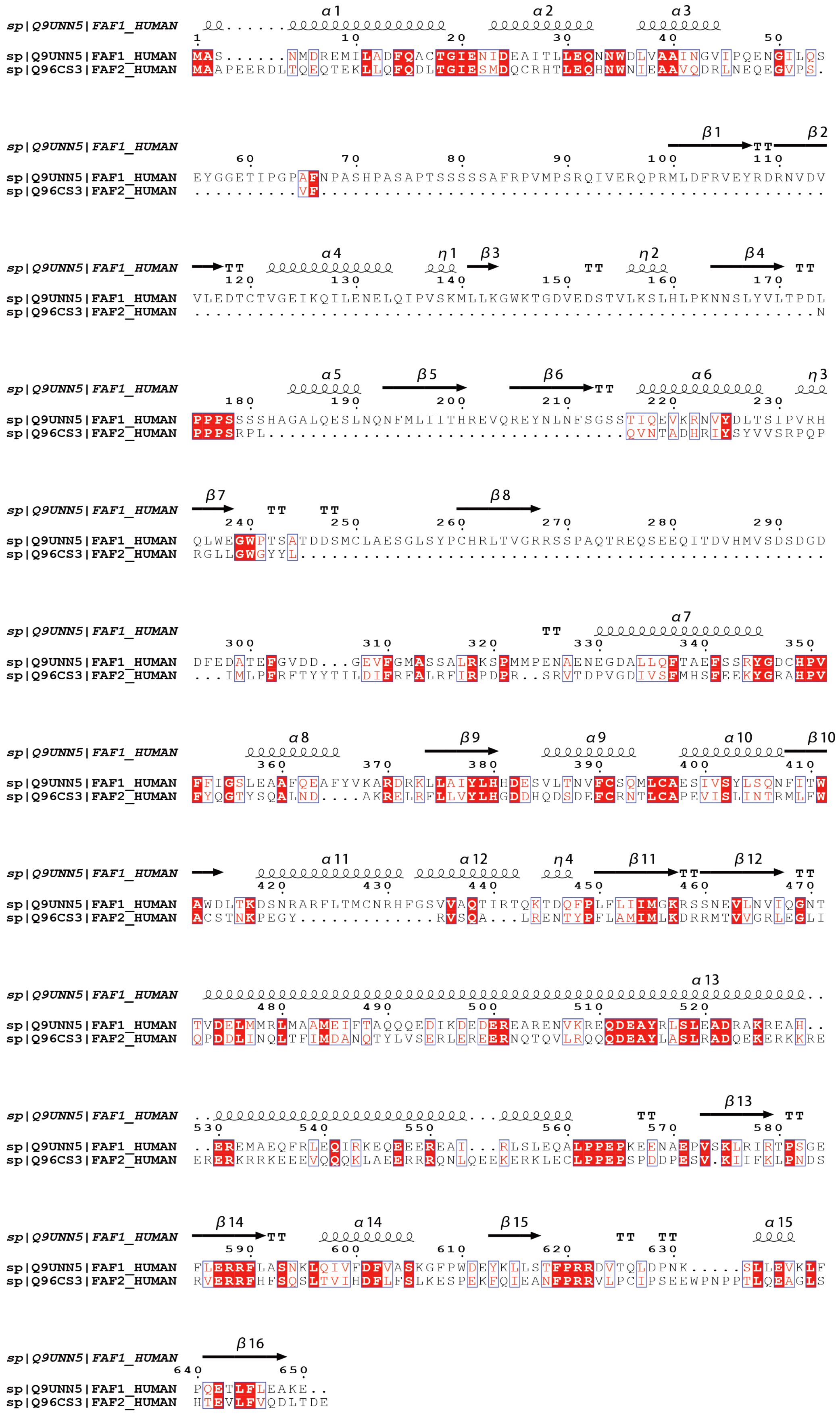

Supplemental Figure 13: Sequence alignment of human Faf1 and Faf2.

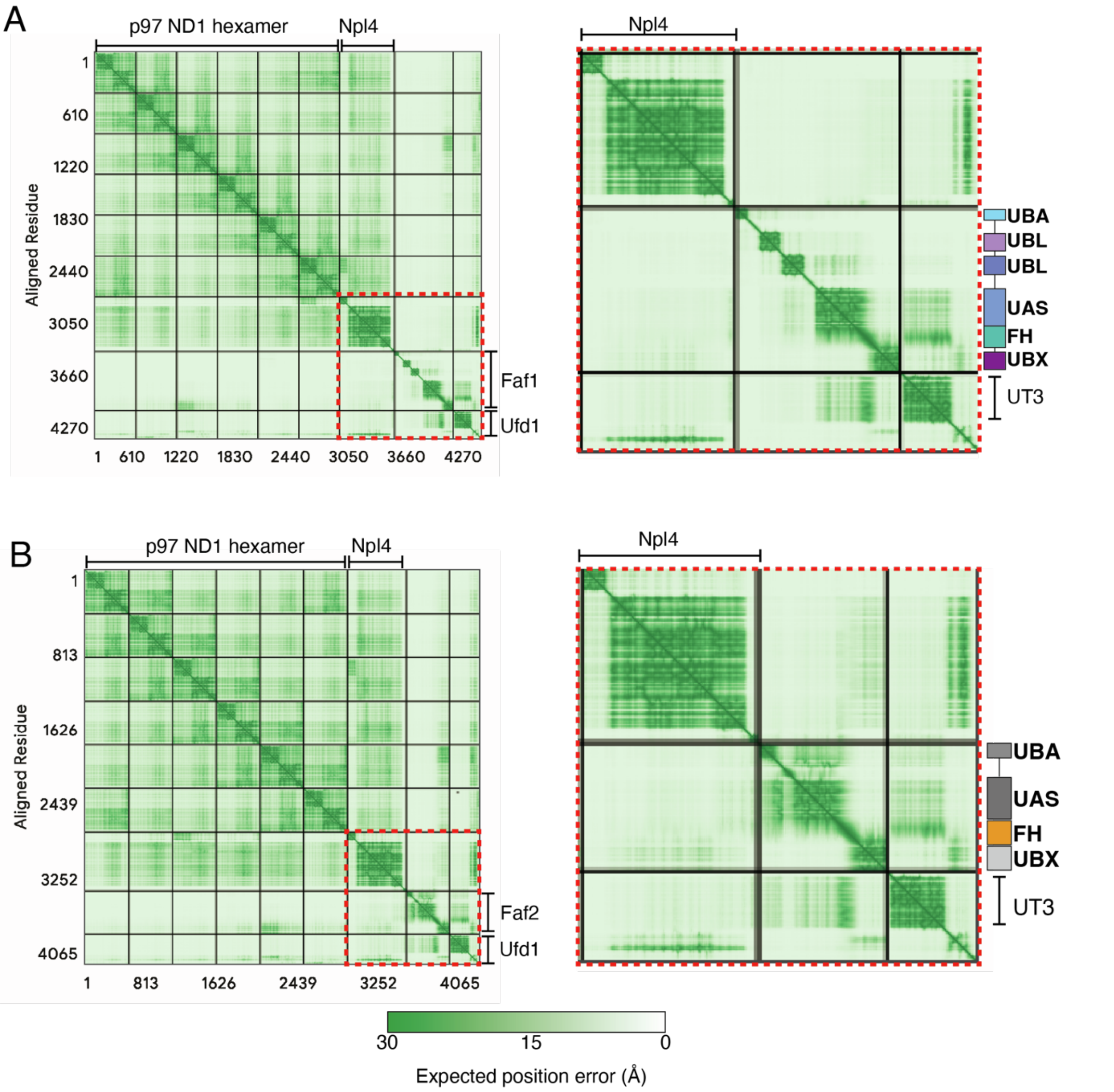

Supplemental Figure 14: Predicted align error (PAE) for the AlphaFold 3 models of the p97-UN-Faf complexes. A) Left: PAE plot for the entire input of p97 NTD-D1, UN, and Faf1. Right: Zoom-in view of the Faf1-UN interaction, as highlighted by the red dashed box on the left. **B)** Left: PAE plot for the entire input of p97 NTD-D1, UN, and the Faf2 UBA-UAS-FH-UBX fragment. Right: Zoom-in view of the Faf2-UN interaction, as highlighted by the red dashed box on the left.

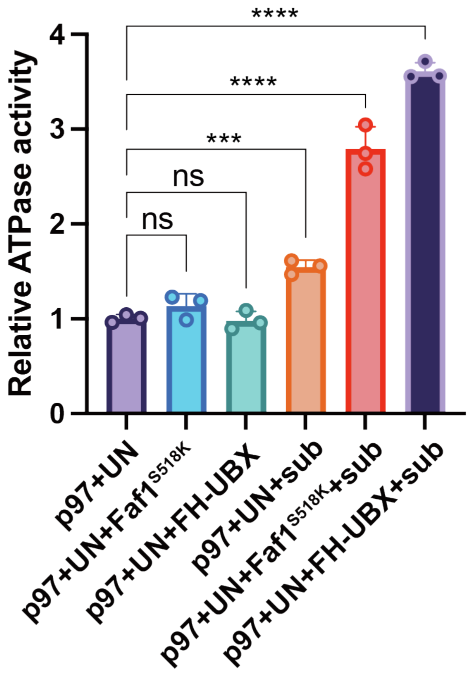

Supplemental Figure 15: Relative ATPase activities of p97-UN (normalized to 1) and its complexes with Faf1^{S518K} of the Faf1 FH-UBX fragment, in the absence or presence of ubiquitinated green Eos substrate. Shown are the mean values and standard deviations of the mean for three technical replicates. Statistical significance was calculated using a one-way ANOVA test: ****p<0.0001; ***p<0.001; ns, p>0.05.

	p97-Ufd1-Faf1 complex (NTD focused)	Ubiquitin-engaged p97-UN-Faf1^{FH-UBX} complex (I state)	Ubiquitin-engaged p97-UN-Faf1^{FH-UBX} complex (Npl4 local)	p97-UN-Faf1^{FH-UBX} complex (PI state)	Ubiquitin-engaged p97-UN-Faf1^{FH-UBX} complex (IC1)	Ubiquitin-engaged p97-UN-Faf1^{FH-UBX} complex (IC2)
EMDB entry<br>PDB entry	EMD-73536<br>9YW2	EMD-76028<br>11TA	EMD-76026<br>11SY	EMD-76074<br>11VE	EMD-76053	EMD-76054
**Data collection and processing**						
Microscope and camera	Titan Krios G4, Falcon 4i					
Magnification	×165,000	×105,000	×105,000	×105,000	×105,000	×105,000
Voltage (kV)	300					
Data acquisition software	SerialEM					
Electron exposure ($e^-/Å^2$)	50					
Defocus range (μm)	-0.8 to -1.6					
Pixel size (Å)	0.743	1.182	1.182	1.182	1.182	1.182
Symmetry imposed	C1					
Initial particle images (no.)	4,705,489	3,956,119				
Final particle images (no.)	943,368	102,875	20,565	99,404	19,930	19,908
Map resolution (Å)	3.27	3.58	4.28	3.85	5.02	5.24
FSC threshold	0.143					
**Refinement**						
Initial model	AlphaFold 3	AlphaFold 3, 7JY5	AlphaFold 3, 8DAW	AlphaFold 3		
Model resolution (Å)	3.58	3.58	4.91	4.08		
FSC threshold	0.5					
Map sharpening *B* factor ($Å^2$)	143.9	67.2	84.5	73.1		

**Model compositions**				
Non-hydrogen atoms	2610	37912	6018	35403
Protein residues	323	4773	755	4663
Ligands	0	ATP: 3, ADP: 9, ZN: 2	ZN:2	ATP: 3, ADP: 9, ZN: 2
***B* factors (Å^2)**				
Protein	158.39	101.32	206.81	126.98
Ligand	N/A	87.18	250.71	92.01
**R.M.S. deviations**				
Bond lengths (Å)	0.003	0.006	0.002	0.003
Bond angles (°)	0.761	0.724	0.593	0.686
**Validation**				
MolProbity score	2.43	2.54	2.01	2.26
Clashscore	16.99	11.42	13.45	9.18
Poor rotomers (%)	3.09	4.76	0	3.17
**Ramachandran plot**				
Favored (%)	95.27	92.94	94.48	94.19
Allowed (%)	4.10	6.86	5.52	5.73
Disallowed (%)	0.63	0.17	0	0.09

Supplemental Table 1: Cryo-EM data collections, refinement and validation statistics, and atomic models for the p97-Ufd1-Npl4 complex with full-length Faf1 and a ubiquitinated substrate or the Faf1 FH-UBX fragment in the presence of free ubiquitin chains.
